# Correlating Synthesis, Structure, and Thermal Stability of CuBi Nanowires for Spintronic Applications by Electron Microscopy and *in Situ* Scattering Methods

**DOI:** 10.1021/acsnano.5c09560

**Published:** 2025-12-02

**Authors:** Alejandra Guedeja-Marrón, Henrik Lyder Andersen, Gabriel Sánchez-Santolino, Lunjie Zeng, Alok Ranjan, Inés García-Manuz, François Fauth, Catherine Dejoie, Eva Olsson, Paolo Perna, Maria Varela, Lucas Pérez, Matilde Saura-Múzquiz

**Affiliations:** † Departamento de Física de Materiales, Facultad de Ciencias Físicas, 16734 Universidad Complutense de Madrid, Madrid 28040, Spain; ‡ Instituto Pluridisciplinar, 16734 Universidad Complutense de Madrid, Madrid 28040, Spain; § 69570 Instituto de Ciencia de Materiales de Madrid (ICMM), CSIC, Madrid 28049, Spain; ∥ Department of Physics, 11248 Chalmers University of Technology, Gothenburg 41296, Sweden; ⊥ 202533 IMDEA Nanociencia, Madrid 28059, Spain; # Departamento de Física de la Materia Condensada (IFIMAC), Universidad Autónoma de Madrid, 28049 Madrid, Spain; ∇ CELLS-ALBA Synchrotron, Barcelona 08290, Spain; ○ 55553 European Synchrotron Radiation Facility (ESRF), Grenoble 38000, France

**Keywords:** nanowires, CuBi, electrodeposition, STEM, powder X-ray diffraction, Rietveld analysis, PDF

## Abstract

Bi-doped copper (Cu_1–*x*_Bi_*x*_) nanowires (NWs), promising candidates for spintronic applications due to their potential for a giant spin Hall effect (SHE), were synthesized, and their structural properties and thermal stability were investigated. Using template-assisted electrodeposition, Cu_1–*x*_Bi_*x*_ nanowires with varying bismuth (Bi) content (*x* = 0, 2, 4, and 7%) and different crystalline domain sizes were fabricated. Structural analysis by advanced electron microscopy and X-ray scattering techniques revealed the influence of synthesis conditions on the resulting NW crystal structure and microstructure, including Bi localization (within the lattice or in the grain boundaries), crystallite domain dimensions, and lattice distortions. While NWs with larger crystalline domains allow homogeneous Bi incorporation into the Cu lattice, NWs with smaller crystalline domains exhibit noticeable Bi accumulation at grain boundaries. The thermal stability of the NWs was examined using variable temperature X-ray diffraction and total scattering. Upon heating, lattice distortions consistent with Bi diffusion out of the Cu lattice were observed, with subsequent crystallization of rhombohedral metallic Bi upon cooling. Microstructural analysis of NWs post heating shows that the recrystallized rhombohedral Bi accumulates in localized regions within the NWs, most likely corresponding to grain boundaries. In some cases, the exsolution of Bi from these regions leads to wedge-shaped fractures in the NWs and the formation of independent Bi particles. These findings establish a foundation for optimizing the SHE performance of Cu_1–*x*_Bi_*x*_ nanowires for spintronic devices by correlating synthesis parameters with microstructural features and thermal behavior.

## Graphical abstract

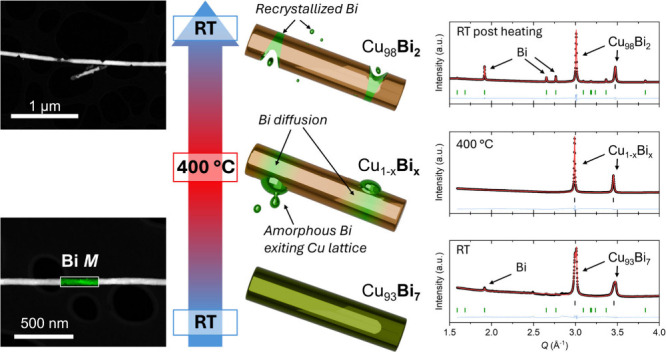

## Introduction

Bismuth (Bi) possesses one of the largest spin–orbit interactions (SOI) of all atoms. Consequently, Bi has been incorporated into semiconductor heterostructures to leverage the strong SOIs induced by Bi for efficient spin control of electrons in semiconductor channels.^1,2^ In the case of metals, significant effort has been made to utilize the strong SOI for producing spin-charge interconversion (SCI),^3^ by incorporating Bi layers in spintronics devices. Surprisingly, in most experiments to date, the SCI efficiency measured in Bi is smaller than that in Pt, Ta, or W, which have a smaller SOI than Bi. Only recently, large SCI has been measured in Ni/Bi(110) structures, ascribing the lack of previous results to the large anisotropy of the effective g-factor of Bi.^4^ The effect is different when Bi atoms are introduced as dopants in a metallic matrix. In particular, Bi impurities in a Cu host were identified by *ab initio* calculations as the best candidates for all-metallic spin-current generation.^5^ This calculation was experimentally verified by Niimi et al., who reported a substantial spin Hall angle (SHA) of approximately −0.24 in CuBi alloys with ∼0.5% of Bi doping.^6^ The presence of spin Hall effect (SHE) in highly Bi-doped Cu films has been confirmed by direct interface-free X-ray spectroscopy measurements in highly Bi-doped Cu films.^7^ Although the mechanism of skew scattering has been proposed as the main driving force of the observed extrinsic spin Hall effect in CuBi alloys,^8^ either the formation of extremely small clusters or the influence of interface roughness and grain boundaries decorated with Bi atoms may also be responsible for the observed phenomenon.^9^ Other effects linked to the large SCI of Bi atoms in a Cu matrix have been recently reported. These include a spin mixing conductance in CuBi/YIG larger than the one shown by similar Pt/YIG structures,^10^ or the possibility of having large spin–orbit torque efficiency,^11^ reflecting the potential of these new alloys for developing spintronics devices. However, before incorporation into devices, it is crucial to have a deeper understanding of the mechanism behind the SOI-related effects and their correlation with the microstructure of the CuBi alloys. To achieve this, the alloys must be prepared using a synthesis/growth method that allows systematic control of sample characteristics such as composition, crystal quality, microstructure, and cluster formation.^12^ In the case of CuBi alloys, electrochemical deposition has been shown to be a highly effective technique to produce nanowires (NWs) enabling precise control over both their composition and crystallinity.^13^

To elucidate the relationship between grain size and spin transport properties, it is essential to examine systems with crystallite sizes both smaller and larger than the reported spin diffusion length, estimated to range from 300 to 500 nm depending on the temperature.^14–17^ For instance, NWs with smaller grain sizes (<500 nm) provide an opportunity to analyze how grain boundaries act as potential scattering sites for spin currents, most likely reducing SHE efficiency. Conversely, NWs with larger grains (>500 nm) allow spin transport properties in a regime with minimized grain boundary effects to be examined, highlighting the intrinsic properties of the CuBi alloy. Furthermore, understanding and controlling the thermal stability of these NWs is particularly critical for addressing fundamental questions about the performance of these materials under applied current, where a temperature increase due to Joule heating is expected. Thus, unveiling the interplay between the synthesis method, micro/crystal/local structure, Bi doping, as well as potential structural changes that may arise in the system due to heat generation, is crucial for the rational optimization of this class of materials.

In the present work, we examine the structural characteristics of Cu_1–*x*_Bi_*x*_ NWs with different crystallite sizes and Bi contents prepared by electrodeposition. Given its established role in generating a significant SHA, we systematically analyze Cu_1–*x*_Bi_*x*_ NWs with Bi concentrations of 0%, 2%, 4%, and 7% to explore the impact of this heavy atom on the material’s structure. The structural analysis is conducted over multiple length scales (atomic, nano, and micro) using a combination of advanced characterization techniques, including scanning transmission electron microscopy (STEM), electron energy-loss spectroscopy (EELS), variable temperature synchrotron powder X-ray diffraction (SPXRD) with Rietveld analysis, and X-ray total scattering (TS) with pair distribution function (PDF) analysis. We demonstrate how varying Bi concentration and electrodeposition synthesis conditions lead to changes in the microstructure, such as Bi distribution within the NWs, crystallite size, and lattice distortions. Furthermore, we conduct an in-depth investigation of the structural response of these systems upon heating using *in situ* variable temperature scattering experiments. While *ex situ* techniques often fail to capture the true behavior of materials under real-world operating conditions, *in situ* methods can provide real-time insights into e.g. phase transitions, atomic diffusion, and strain-induced effects taking place when the material is subjected to real world operating conditions. The ability to fabricate Cu_1–*x*_Bi_*x*_ NWs with tailored structural characteristics and stability opens new avenues for designing materials with optimized SHE. By understanding the structure and thermal stability of high-quality electrodeposited Cu_1–*x*_Bi_*x*_ NWs, we establish a foundation for the precise fabrication of NWs with enhanced SHE and optimized spin transport properties for the development of next-generation spintronic devices.

## Results and Discussion

### Morphology and Microstructure of NWs: STEM/4D-STEM

Bi-doped Cu NWs with about 50 nm in diameter and varying amounts of Bi content (Cu_1–*x*_Bi_*x*_ with *x* = 0, 0.02, 0.04, 0.07) were synthesized by template-assisted electrodeposition within the pores of anodized aluminum oxide (AAO) templates as described in the methods section.^18^ Notably, the length of the synthesized NWs is determined by the growth time, while the diameter of the NWs is determined by the pore diameter of the AAO template resulting from the anodization process. As we have previously reported,^13^ the growth of polycrystalline NWs with different crystalline domain sizes can be achieved by tuning the overpotential during electrodeposition. It has been shown that faster deposition rates generally lead to smaller crystallites due to enhanced nucleation,^19^ whereas slower rates favor growth of larger crystallites.^20^ Reducing the overpotential during the growth leads to a lower current density, producing a more uniform and consistent electrodeposition, which results in improved crystallinity.^21^ However, employing low current densities may also affect the conductivity of the electrolyte,^22^ and can prevent deposition from taking place. In order to lower the electrodeposition rate without reducing the conductivity of the electrolyte, an alternative approach is needed. Chelating agents, such as acids, are often used to enhance the solubility of metallic ions, due to their ability to form complexes with metals.^23^ We believe the formation of such metallic complexes may also be a handle to tune the growth rate by slightly “hindering” the electrodeposition of the metals and, therefore, reducing the crystallization speed. If effective, different concentrations of chelating agents could lead to different crystallite sizes in the obtained NWs. In this study, tartaric acid (TA) was used as chelating agent.^24^ During the synthesis process, TA was incorporated into the electrolyte to improve Bi solubility, allowing a better tuning of the crystallite sizes in the Cu_1–*x*_Bi_*x*_ NWs.^25^ Electrodeposition growth curves showed that NWs synthesized with lower TA concentrations exhibit a growth time of 7500 s, whereas those with higher TA concentrations require 12000 s to complete growth. However, despite the change in electrodeposition rate due to TA, the current densities were on the same order of magnitude in both cases, suggesting that the same amount of Bi is being incorporated into the NWs, but at a slower electrodeposition rate in the case of high TA concentration. These results indicate that the use of TA effectively reduces the electrodeposition rate.

A total of seven Cu_1–*x*_Bi_*x*_ samples were prepared using different concentrations of Bi­(NO_3_)_3_ and TA. An overview of the synthesized samples is given in Table S1. The notation SC and LC refers to small crystallites and large crystallites, respectively, and the number given beside it describes the percentage of Bi in the resulting Cu_1–*x*_Bi_*x*_ NWs as determined by STEM-EELS (described later). Samples of three different nominal concentrations were prepared for both the SC and LC type of NWs. These are referred to as SC2, SC4, SC7, LC2, LC4, and LC7, indicating whether they form NWs with small or large crystallites, and the approximate mean Bi content (2%, 4%, and 7%). Two additional reference samples of pure Cu and pure Bi NWs were also synthesized. Additionally, a sample with 7% Bi and an even higher amount of TA than the rest of the LC samples (denoted as LC7*) was synthesized to further examine the influence of TA on the crystallite size of the NWs.

Figure 1a,b shows representative low magnification STEM images of NWs released from the alumina template for the SC7 and LC7* samples, which were synthesized with low and high (four times higher) concentrations of TA, respectively (see Table S1 for sample details). The samples consist of NWs with average lengths of around 10 μm, as the cross-sectional SEM view of the NWs embedded in the AAO template depicts (see Figure S1). However, in the STEM images some NWs were found to be shorter, likely due to local fractures suffered during the release from the template and/or the preparation for STEM measurements.

**Figure 1:**
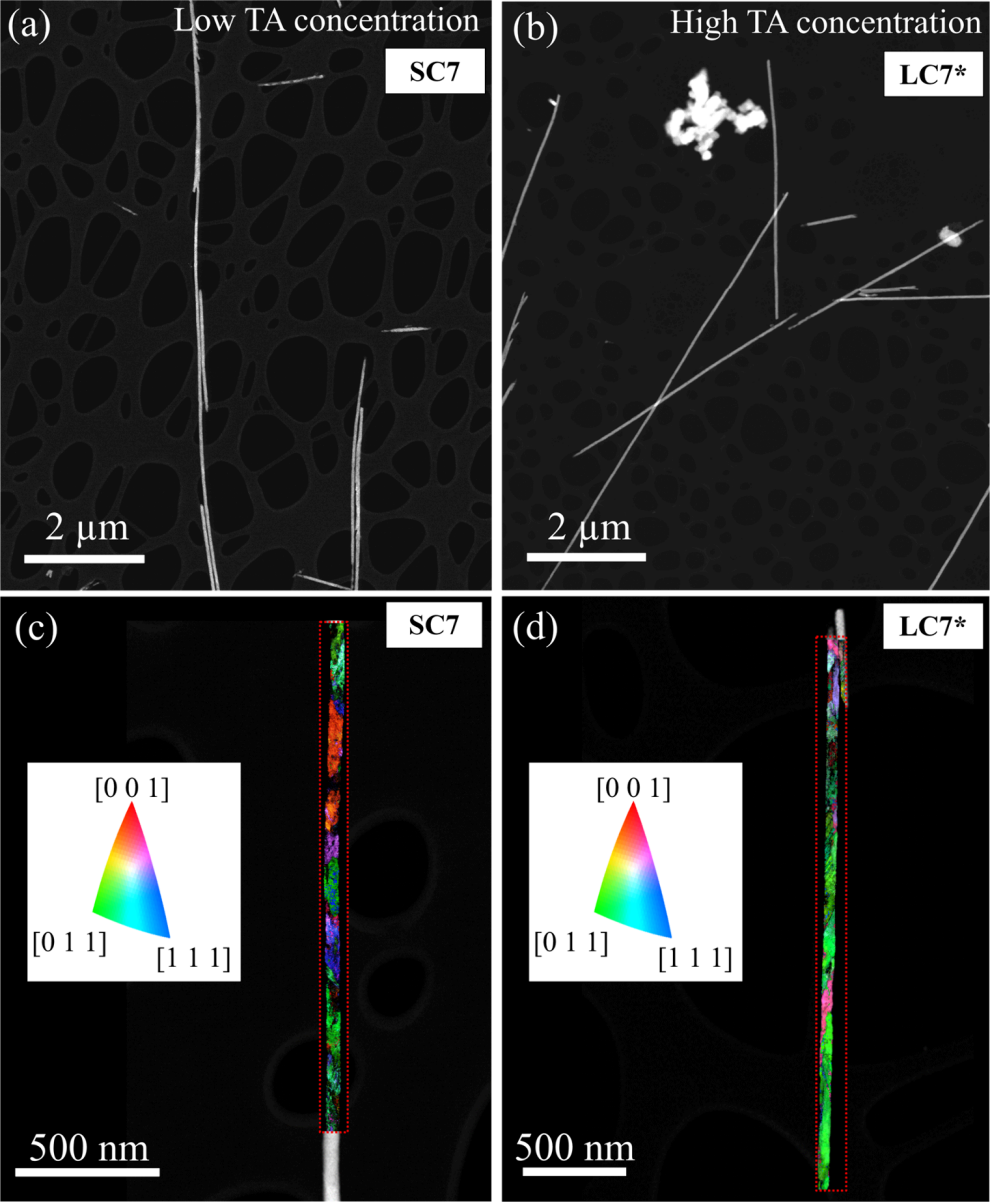
(top panels) High-angle annular dark field (HAADF) low magnification STEM images of Bi-doped Cu NWs synthesized with (a) a low concentration (SC7) and (b) a high concentration (LC7*) of TA. (bottom panels) Crystal orientation maps derived from 4D-STEM datasets of (a) SC7 NW and (b) LC7* NW showing crystalline domains and out-of-plane crystalline orientation colored by the stereographic projection of the [001], [011], and [111] directions.

To investigate the effect of TA concentration on the resulting sizes of the crystalline domains of the Cu_1–*x*_Bi_*x*_ NWs, four-dimensional STEM (4D-STEM) spatially resolved nanodiffraction measurements were performed to obtain crystal-orientation maps.^26^ Figure 1c, d shows the crystal orientation maps of two NWs from samples SC7 and LC7*, grown from electrolytes with different TA concentration: SC7 (0.33 M TA), LC7* (1.32 M TA) (Table S1). The maps show a clear difference in the crystallite sizes of the samples. The SC7 NW (lower TA concentration) shows smaller crystallites of approximately 200 nm, whereas LC7* (higher TA concentration) shows much larger crystallites up to around 1 μm. To systematically investigate the effect of TA concentration, crystallite sizes were extracted from 4D-STEM orientation maps acquired for multiple nanowires across both the SC (low TA concentration) and LC (high TA concentration) series, from which histograms representing the grain size distributions of the different samples were constructed (see Figure S2). The analysis demonstrates that increasing the TA concentration leads to larger crystallite grains irrespective of Bi content. The LC and LC* nanowires consistently exhibit grain sizes exceeding 500 nm, whereas the SC nanowires remain well below this threshold. Notably, when comparing nanowires synthesized with identical TA concentrations but different Bi contents (i.e., SC2 vs SC7 and LC2 vs LC7), the histograms indicate that a higher Bi content slightly reduces the average grain size and narrows the size distribution. Given these results, the samples synthesized with low concentration of TA are referred to as small crystallite-size (SC) nanowires, and those synthesized using higher TA concentration are referred to as large crystallite-size (LC) nanowires.

### Composition and Bi Distribution: EELS/EDS

To determine the nominal Bi content within the grains of the different Cu_1–*x*_Bi_*x*_ NWs, EELS measurements were conducted on the NWs grown with varying Bi­(NO_3_)_3_ concentrations in the precursor electrolyte (see Table S1). Figure 2 illustrates the observed spatial distribution of the relative Bi atomic percent within the grains for three NWs samples in the LC series (high TA concentration) grown using different Bi­(NO_3_)_3_ concentrations in the electrolyte. The Bi distribution was examined using EELS, and maps of the relative Bi content (in atomic percent) for each NW were obtained using the Gatan Digital Micrograph standard routines. Parts a–c of Figure 2 display the high angle annular dark field (HAADF) images of the areas sampled for samples LC2, LC4, and LC7, respectively. White rectangles mark the regions where EEL spectrum imaging was performed on each specimen. Cu *L*_2,3_ and Bi *M*_4,5_ absorption edges were studied in order to extract the relative Cu/Bi compositions. Figure 2d–f displays the resulting Bi relative compositional maps for each material, again showing data for LC2, LC4, and LC7 from left to right. Each panel exhibits two maps that correspond to the same data adjusted to different contrast. The Bi relative composition appears inhomogeneous, so contrasts have been manually tuned to visually highlight the spatial compositions within the inner core (marked with blue rectangles) and the outer shell (highlighted with green rectangles). Histograms for each dataset have been constructed for statistical quantification, as shown in Figure 2g–i. Two main contributions are present in all maps, associated with the core and the native oxide layer on the surface of the NWs, formed due to air exposure.^27–29^ To distinguish the two contributions, the histograms were fitted using a superposition of two Gaussian curves, highlighted in blue (core) and green (shell). In all cases, a significant Bi segregation, in the form of a few nm thick Bi oxide shell where the local Bi concentration doubles with respect to the core, is detected (O map show in Figure 3c). All cores exhibit a relatively homogeneous Bi distribution within the grains, with a direct correlation between the concentration of Bi­(NO_3_)_3_ in the electrolyte, and the resulting Bi atomic percentage in the NWs, with average Bi compositions of 1.4%, 4.9%, and 7.6%, respectively, for the three samples (with a 1% uncertainty in all cases). The EELS maps therefore confirm accurate control over the composition of the Cu_1–*x*_Bi_*x*_ NW cores, consistent with the targeted Bi contents (2%, 4%, and 7%) obtained from the three tested Bi­(NO_3_)_3_ concentrations (2, 4, and 8 mM).

**Figure 2:**
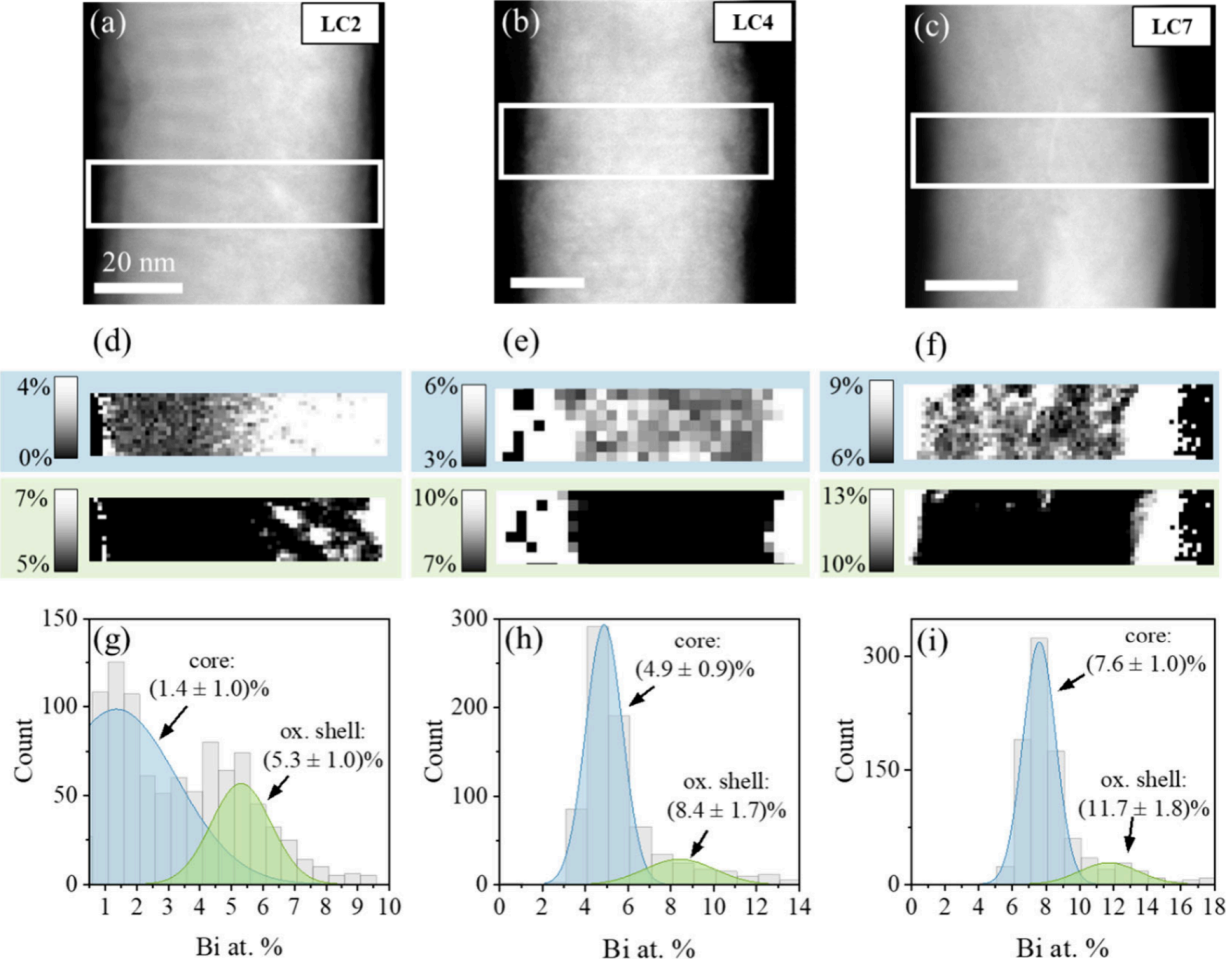
(a–c) ADF-STEM images of single NWs from samples (a) LC2, (b) LC4, and (c) LC7. (d–f) Spatially resolved maps of the relative Bi composition (in atomic %) within the regions marked with white rectangles in parts a–c, respectively. Top (blue rectangle) and bottom (green rectangle) maps in each panel show the same data, with different contrasts adjusted to highlight the contribution of core (blue) and oxide shell (green) regions. (g–i) Corresponding histograms quantifying the Bi content resulting from data in (d) LC2, (e) LC4, and (f) LC7 NWs. The two Gaussian curves fitted to the EELS histograms correspond to the Bi contributions from the core (blue) and shell (green) regions.

**Figure 3:**
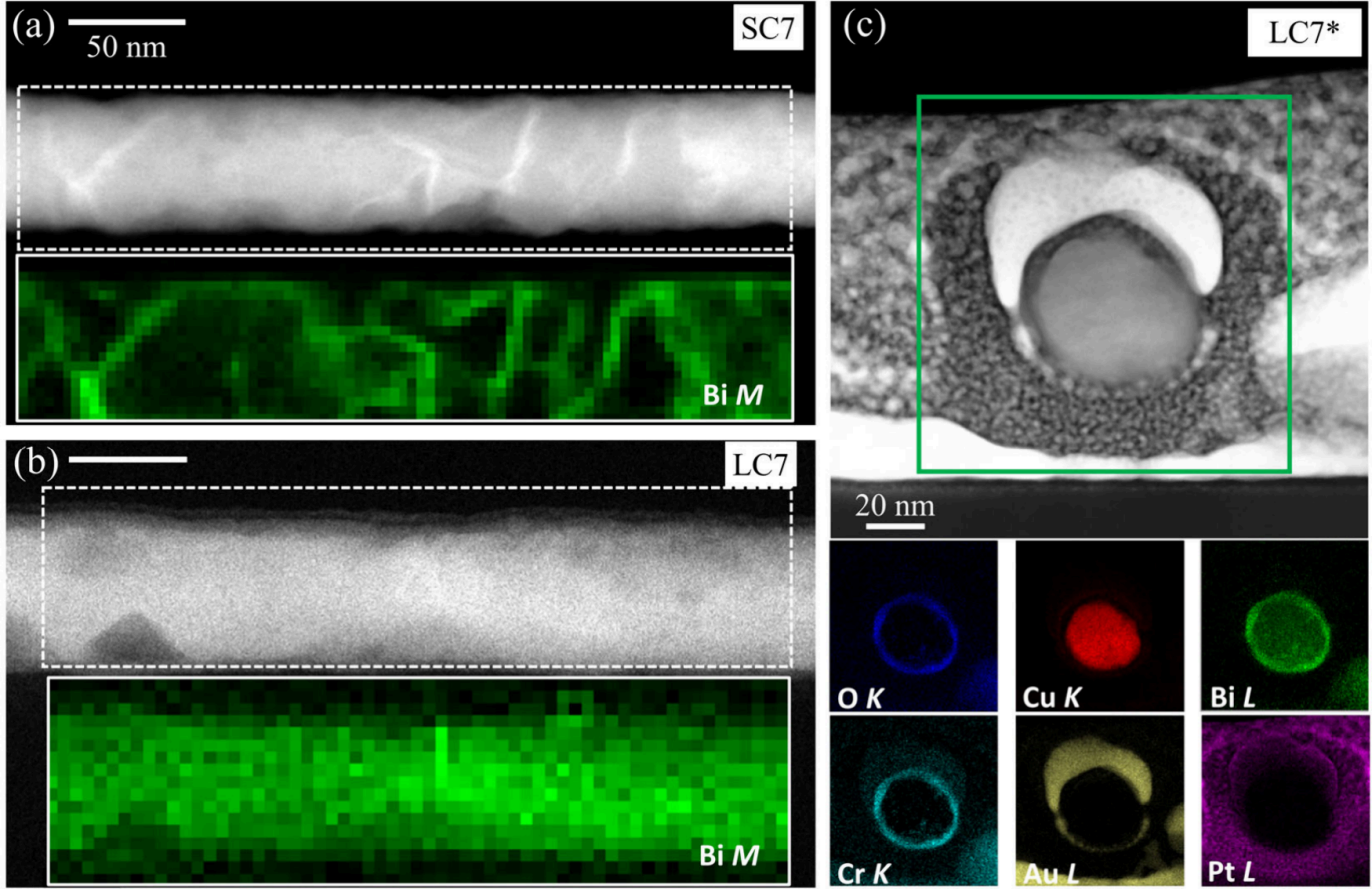
(a,b) ADF-STEM low magnification images of (a) a SC7 NW and (b) a LC7 NW, with inserts below showing the Bi *M*_4,5_ edge EELS signal (green) extracted from the white squared regions. (c) HAADF image of the cross-section view of a LC7* NW and corresponding EDS elemental maps derived from the O *K*, Cu *L*, Bi *L*, Cr *K*, Au *L*, and Pt *L* edges from top to bottom and left to right, respectively.

For comparison between the SC and LC series, EELS maps were also collected on both the SC7 and LC7 NW samples. Figure 3a,b displays low magnification HAADF images (top panels) of SC7 (a) and LC7 (b) NWs, along with maps showing the integrated signal under the Bi *M*_4,5_ edge in a false color scale extracted from EEL spectrum images, measured within the regions highlighted with white rectangles for both samples (bottom panels). These elemental maps indicate that Bi is not distributed equally within the grains of the two types of NWs. For the SC7 sample, Bi accumulates preferentially in the grain boundaries. Meanwhile, for the LC7 sample, where larger grains are formed and less grain boundaries are present, Bi is distributed homogeneously throughout the grains. These results indicate that tuning the synthesis method, not only provides a handle for controlling the crystallite sizes of the NWs, but also leads to significant differences in the distribution of the Bi dopant within them. This difference in Bi distribution may lead to variations in the spin length diffusion of the NWs, which in turn would influence the SHE response.^8^

To further investigate the spatial homogeneity of the Bi radial distribution within the NWs, a cross-section of a few LC7* NWs dispersed onto a Si substrate was prepared by FIB-SEM (Figure 3c and Figure S3). After drop-casting the NWs on a Si substrate and before the FIB-SEM process, the NWs were coated with a layer of Au using magnetron sputtering to prevent ion damage. In Figure 3c, an ADF image of the cross-section of one of the LC7* NWs is shown. The NW core and shell regions are clearly defined. The bottom panels depict a series of elemental maps obtained by energy dispersive X-ray spectroscopy (EDS), including all O *K*, Cu *L*, Bi *L*, Cr *K,* Au *L*, and Pt *L* edges of interest. The Cu core is well-defined, with a significant Bi signal that is homogeneously distributed in the radial direction. The fact that both the Bi and Cu signals are homogeneously distributed throughout the NW core, verifies the effective insertion of Bi within the Cu lattice and the lack of discrete Bi clusters. A Bi oxide shell is also clearly detected, as well as some Cr contamination. Such surface layers of Cr and Bi oxides are a few nm thick at most, and are a result of the chemicals (H_2_CrO_4_ solution) used during the release of the NWs from the nanoporous AAO template and the oxidation due to exposure to air. The Au and Pt protective layers derived from the conventional lamella preparation by the FIB-SEM method are also identified around the NWs.

### Crystal Structure: Room Temperature (RT) SPXRD

To investigate the average crystal structure of the samples and whether secondary oxide phases or metallic Bi clusters are formed in the as-synthesized NWs (still embedded in the AAO template), high-angle resolution synchrotron powder X-ray diffraction (SPXRD) data were collected for SC and LC samples with different Bi doping (see Table S1). Figure 4 shows the Rietveld refinement of SPXRD data collected at room temperature (RT) on NWs from the two series (SC and LC) with different Bi doping levels (0%, 2%, 4%, and 7%). For reference, and given that different unit cell parameters are often obtained for nanosized and bulk structures of the same compounds, a sample of pure Cu NWs (0% doping) and a sample of pure Bi NWs were also grown using the same method as for the Bi-doped Cu samples. The refined RT lattice parameter of our synthesized Cu NWs was found to be 3.61446(1) Å, which is slightly smaller than that reported for bulk, defect free Cu of 3.61491 Å at 25 °C.^30^ It is worth noting that metallic Cu and Bi do not crystallize in the same structure. While Cu crystallizes in the cubic *Fm*-3*m* space group, metallic Bi crystallizes in the rhombohedral *R-3m* structure. Illustrations of the Cu and Bi crystal structures are shown in Figure S4, and the Rietveld refinement of SPXRD data collected on the pure Bi NWs sample is given in Figure S5. In the refinements, the atomic positions and occupancies were kept fixed for both phases, while scale factors, unit cell parameters, zero shift, and an overall isotropic thermal parameter (*B*_iso_) were refined. The peak profiles were modeled using the Thompson–Cox–Hastings formulation of the pseudo-Voigt function, using a platelet-vector-size model.^31^ Notably, the instrumental contribution to the total peak broadening was determined by refinement of data collected on an NIST LaB_6_ 660b calibrant in the same instrumental configuration and deconvoluted from the sample broadening in the refinements. Since the Cu structure contains just a single Wyckoff site, the amount of incorporated Bi within the lattice cannot be extracted from the SPXRD data, as the occupancies of different elements (although they have different scattering factors) on the site are fully correlated with the scale factor.^32^ Therefore, all Cu_1–*x*_Bi_*x*_ phases were refined as pure Cu. Although this may lead to a slight error in the refined weight fractions of Bi-rich, Bi-poor and metallic Bi phases (see discussion later), the effect is minor due to the small percentage of Bi present in the samples, leading to a potential error of approximately 2% in refined weight fractions (see Table S2). Notably, the peak shapes of the secondary nanosized metallic Bi phase were fitted assuming spherical strain-free crystallites.

**Figure 4:**
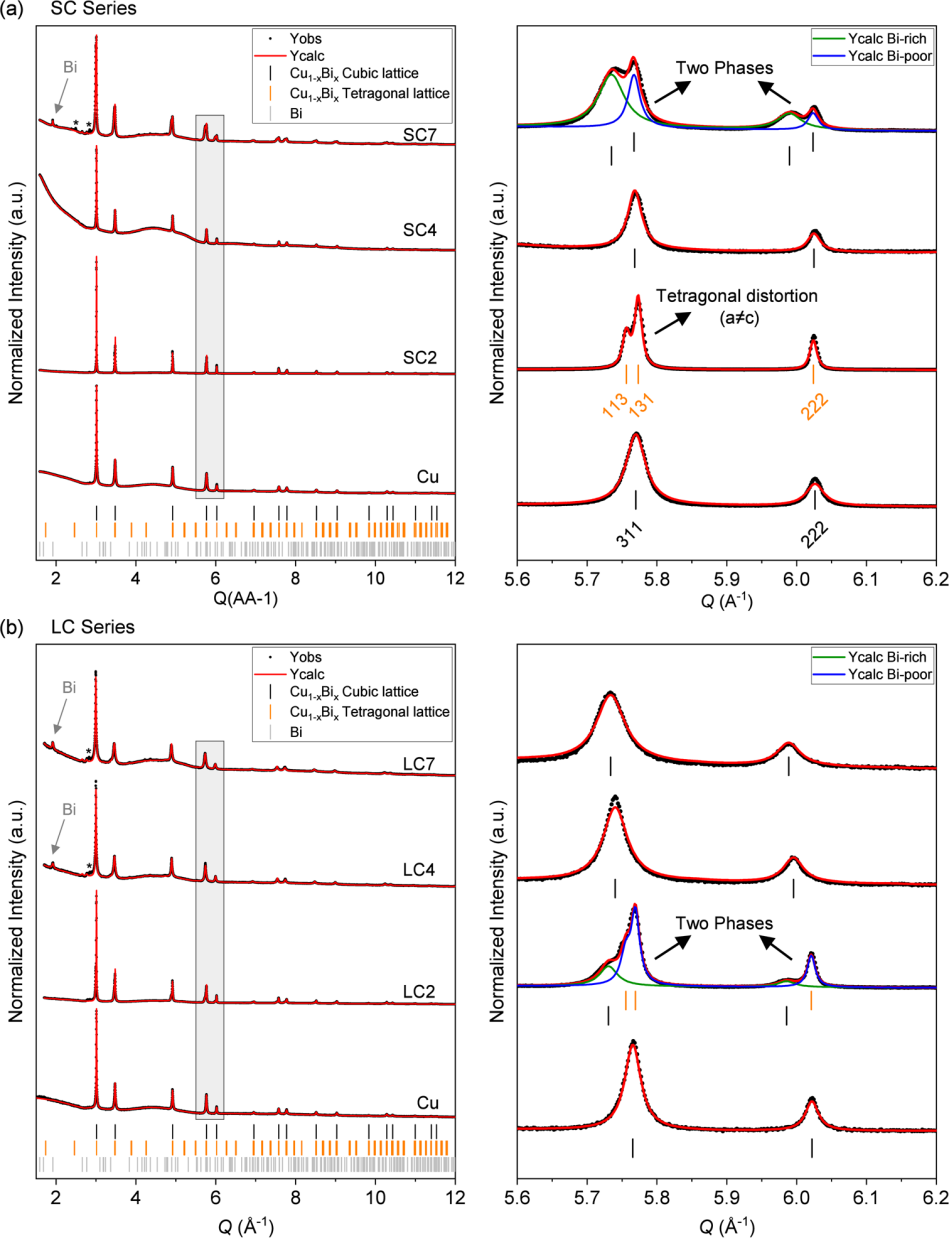
Room temperature powder X-ray diffraction patterns and corresponding Rietveld fits for the (a) SC and (b) LC sample series of nanowires with different Bi doping concentrations (0%, 2%, 4%, and 7%). The positions of the Bragg peaks of the cubic and tetragonal Cu_1–*x*_Bi_*x*_ structures are given by black and orange bars, respectively, and in gray bars for rhombohedral Bi. When two phases are present, the calculated model for each phase is given by green and blue lines on the right-hand-side panels. The Miller indices of the cubic and tetragonal phases shown in the right-hand-side panel of (a) are given to illustrate the peak splitting taking place in the tetragonally distorted lattice. Minor peaks corresponding to unknown impurities (*Q* ∼ 2.9 Å^–1^) are marked with an asterisk.

The high-angular resolution SPXRD data revealed that, in the SC series (Figure 4a), the sample with 2% Bi (SC2) exhibits a tetragonal distortion of the lattice with *a* = *b* = 3.61001(1) Å and *c* = 3.62468(1) Å. This is clearly observed by the splitting of, e.g., the 200 reflection (at *Q* ∼ 3.48 Å^–1^) into two distinct reflections (002 and 020), and the 311 reflection (at *Q* ∼ 5.77 Å^–1^) into two distinct reflections (113 and 131) as observed in the enhanced *Q*-regions on the right in Figure 4a. This tetragonal distortion is not observed for the SC4 or SC7 samples, which are both cubic. However, a consistent splitting of all diffraction peaks is observed for the sample with the highest Bi content (SC7). This consistent splitting does not correspond to a tetragonal distortion, as observed for SC2. Rather, in SC7, the cubic *Fm*-3*m* lattice splits into two cubic phases of different unit cell volumes. The presence of the two cubic phases suggests the formation of two Cu_1–*x*_Bi_*x*_ alloys of different compositions, one being Bi-poor and another one being Bi-rich. The larger sublattice phase, most likely being Bi-rich (considering the atomic radii of Cu = 1.35 Å vs Bi = 1.60 Å),^33^ constitutes the majority of the sample (70.4(5) wt %). It also exhibits a larger broadening of the peaks, indicating smaller crystalline domains and/or higher strain of the Bi-rich phase compared to those of the Bi-poor phase. Notably, the Bi-rich phase remains cubic rather than exhibiting the rhombohedral structure of metallic Bi. In addition, a small amount of metallic Bi in rhombohedral *R*‑3*m* (1.75(8) wt %) was also observed in the SC7 sample (see Figure S6), indicating that some metallic Bi clusters also formed during the electrodeposition process.

Figure 4b shows the Rietveld refinement of the SPXRD data from the samples in the LC series. Here, the higher Bi content NWs (LC4 and LC7) consist of a single homogeneous cubic phase, while the LC2 sample shows the presence of two cubic crystalline phases with different unit cell volumes (similar to the observation for SC7). Furthermore, careful inspection of the data reveals that the major Cu_1–*x*_Bi_*x*_ phase in the LC2, which constitutes 66.9(4) wt %, also exhibits a tetragonal distortion of the lattice equivalent to the one observed in the SC2 sample, with a unit cell of *a* = *b* = 3.61108(1) Å and *c* = 3.62295­(3) Å. The minority phase (33.1(4)%) was refined as cubic with *a* = *b* = *c* = 3.63640(4) Å. The refined weight fractions, unit cell parameters, and unit cell volumes of all samples are given in Table S3.

The refined unit cell volume of samples from both the SC and LC series is plotted in Figure 5 as a function of Bi content obtained from the EELS data. For samples where two Cu_1–*x*_Bi_*x*_ phases are present (LC2 and SC7), both of the refined unit cell volumes are shown in green and blue for the Bi-rich and Bi-poor phases, respectively, as well as the weighted average unit cell volume in black. In those cases, the weight percentages of the two phases are also given. As Figure 5 indicates, the LC series exhibits a gradual, almost linear increase in the average unit cell volume with increasing Bi content (considering the weighted average unit cell volume in LC2). This is consistent with Vegard’s law,^34^ and confirms effective Bi incorporation into the Cu lattice for the entire sample (not only in isolated NWs). In contrast, for the SC series, minimal lattice expansion with increasing Bi content is observed for the SC2 and SC4 samples as well as for the minority phase of SC7 (i.e., the Bi-poor phase, marked in blue). The main phase of SC7 (Bi-rich, green), however, exhibits a unit cell volume closer to that of the LC7 sample.

**Figure 5:**
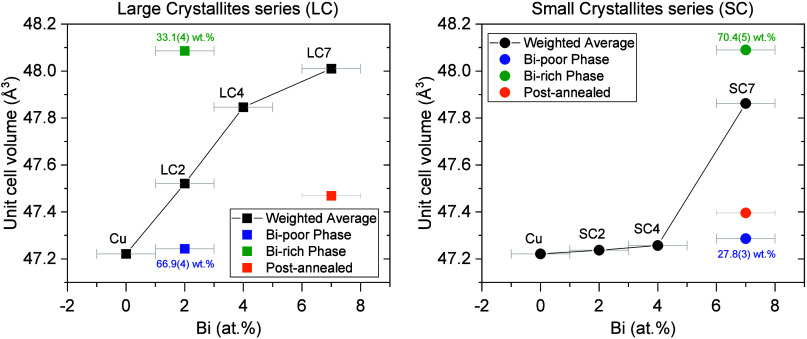
Refined unit cell volumes as a function of Bi content (extracted from EELS data) for the large-crystal (LC) and small-crystal (SC) series. For LC2 and SC7, where two phases were refined, the unit cell volume of both phases is given in green and blue, with the black symbol corresponding to the weighted average unit cell volume. The errors on the refined unit cell volumes (error on *Y* axis) are smaller than symbol sizes.

The subtle volume change in the SC2 and SC4 samples corroborates that only a negligible amount of Bi is effectively incorporated into the crystalline lattice, and instead, most of the Bi localizes at the grain boundaries as observed in the EELS data (see Figure 3). The same trend is also followed by the Bi-poor phase of SC7. However, the main phase of SC7 (70.4(5) wt %) does exhibit the expected lattice expansion within the grains due to Bi insertion. This suggests that for sufficiently high Bi concentrations, incorporation of Bi within the grain also takes place for SC NWs. The Bi at the grain boundaries is highly disordered or amorphous and therefore does not give rise to Bragg reflections in the SPXRD data. These results indicate that for low Bi content, the accumulation of Bi in the SC series appears in a noncrystalline, boundary-localized form, contributing little to the average lattice distortion within grains, whereas in the LC samples, homogeneous insertion of Bi into the crystalline lattice leads to a consistent increase in lattice volume with Bi content, both for the low and high Bi content samples. This demonstrates that the STEM+EELS observations are representative of the entire sample and thus indicate that, through the reported synthesis/growth method, it is possible to control the Bi content and its distribution, although further investigation is needed to determine how the crystalline and amorphous Bi distribution influences the SHE of the material.

Notably, the crystallite sizes of the NWs cannot be accurately determined from the SPXRD data in this system. The 4D-STEM data reveal crystallites of several hundred nanometers in size along the length of the NWs, which is above the resolution limit for the X-ray diffraction size determination. While the crystallite diameter falls within the resolution range (approximately 50 nm), extraction of anisotropic size information is unreliable due to the cubic symmetry of the lattice. This symmetry results in equivalent lattice planes along both the nanowire length and diameter, rendering the extraction of trustworthy crystallite sizes from the Rietveld refinements impossible.

### Thermal Stability

Understanding the thermal stability of the studied NWs is essential, as their potential use in spintronic devices involves exposure to electric currents, which can induce significant temperature increases due to Joule heating. Spintronic technologies are still under development and operational parameters such as device geometry, architecture, and thermal constraints are yet to be standardized. Nevertheless, future devices will likely be based on large-scale arrays of metallic nanostructures (e.g., in racetrack memories, logic circuits, or spin-based interconnects), where thermal management becomes a critical design factor. Depending on the specific application and the spintronic effect being harnessed, devices may experience either short, intense current pulses or prolonged current exposure, each with distinct thermal implications. For this reason, we here examine the crystal and microstructural consequences of heating the material by two different approaches, i.e., slow and fast heating, which seek to emulate the induced Joule heating due to long and short pulse currents, respectively.

In applications exploiting current-induced magnetic domain wall motion, such as in three-dimensional spintronic memory elements, devices typically operate under short, nanosecond-scale current pulses with high current densities, often exceeding 10^11^ A/m^2^.^35^ While the average temperature increase in such devices may be limited, transient thermal spikes can be significant. For example, Bran et al. studied metallic nickel nanowires and found that temperatures can transiently exceed 630 K (∼357 °C) under current densities of 8 × 10^11^ A/m^2^, even during short 8 ns pulses.^36^ These findings highlight that even under brief operation, localized Joule heating can reach levels high enough to potentially induce material changes, especially in nanoscale systems where heat dissipation is limited. Though such events are brief, their cumulative impact over repeated cycles may affect material stability, particularly in systems in which impurity segregation or phase transformation is possible. On the other hand, detection of spin–orbit-related phenomena, including the spin Hall effect and/or orbital Hall effect, typically involves steady-state or long-pulse measurements. These effects produce relatively weak electrical signals, requiring high current densities (usually also on the order of 10^10^–10^11^ A/m^2^) to build up detectable spin or orbital accumulations.^7,37–41^ It is therefore essential to understand the thermal stability limits of metallic NWs for spintronic applications such as Cu_1–*x*_Bi_*x*_ NWs, even if they are not expected to operate at sustained elevated temperatures under normal use.

To estimate the potential temperature increases in nonadiabatic conditions, we performed a simulation of Joule heating in a copper nanowire using the software COMSOL Multiphysics (see Figure S7 and additional information in Supporting Information). The results show that attainable temperatures vary strongly with both the current density and exposure time, confirming that long-duration experiments can indeed push device materials toward thermally unstable regimes at current levels commonly used in spintronic measurements.

Here, we probe a wide temperature range (up to 450 °C) using both fast and slow heating protocols as well as sustained heating for long periods of time (20 min) to map the complete landscape of structural transformations in these nanowires. This approach allows us to identify not just the onset of irreversible phase segregation (e.g., Bi crystallization) but also the safe operational window where only reversible changes (e.g., thermal expansion) occur.

### Variable Temperature (VT) SPXRD with Slow Heating and Cooling

To examine the thermal stability of the Cu_1–*x*_Bi_*x*_ NW structure with slow heating, variable temperature SPXRD data were collected on the SC7 sample upon heating to 450 °C and subsequent cooling (see Figure 6a and Figure S8). A heating ramp of 2 °C/min was applied from RT to 450 °C (∼3.5 h) followed by cooling at 20 °C/min (∼0.5 h) back to room temperature, while sequentially collecting diffraction patterns with a time resolution of 1 min. Sequential Rietveld analysis was carried out on the collected data, and the refined unit cell parameters and weight fractions are shown in Figure 6b,c. As discussed previously, two Cu_1–*x*_Bi_*x*_ phases and a minority metallic Bi phase were observed for the SC7 sample. As seen in Figure 6a,b, when the heating starts (in the RT to 100 °C region), the unit cell parameters of both the Bi-rich (∼70 wt %) and Bi-poor (∼30 wt %) Cu_1–*x*_Bi_*x*_ phases increase linearly with the same slope due to thermal expansion. In the case of the Bi-poor phase, this linear expansion behavior continues up to a temperature of approximately 250 °C, at which this phase starts to disappear. Meanwhile, the Bi-rich phase follows an entirely different trend: At approximately 100 °C the increase in the unit cell slows down, reaching a maximum unit cell of 3.64116(4) Å at 143 °C. From that point, the unit cell exhibits negative thermal expansion, reducing in size with increasing temperature up to approximately 300 °C, above which it starts to increase linearly again.

**Figure 6:**
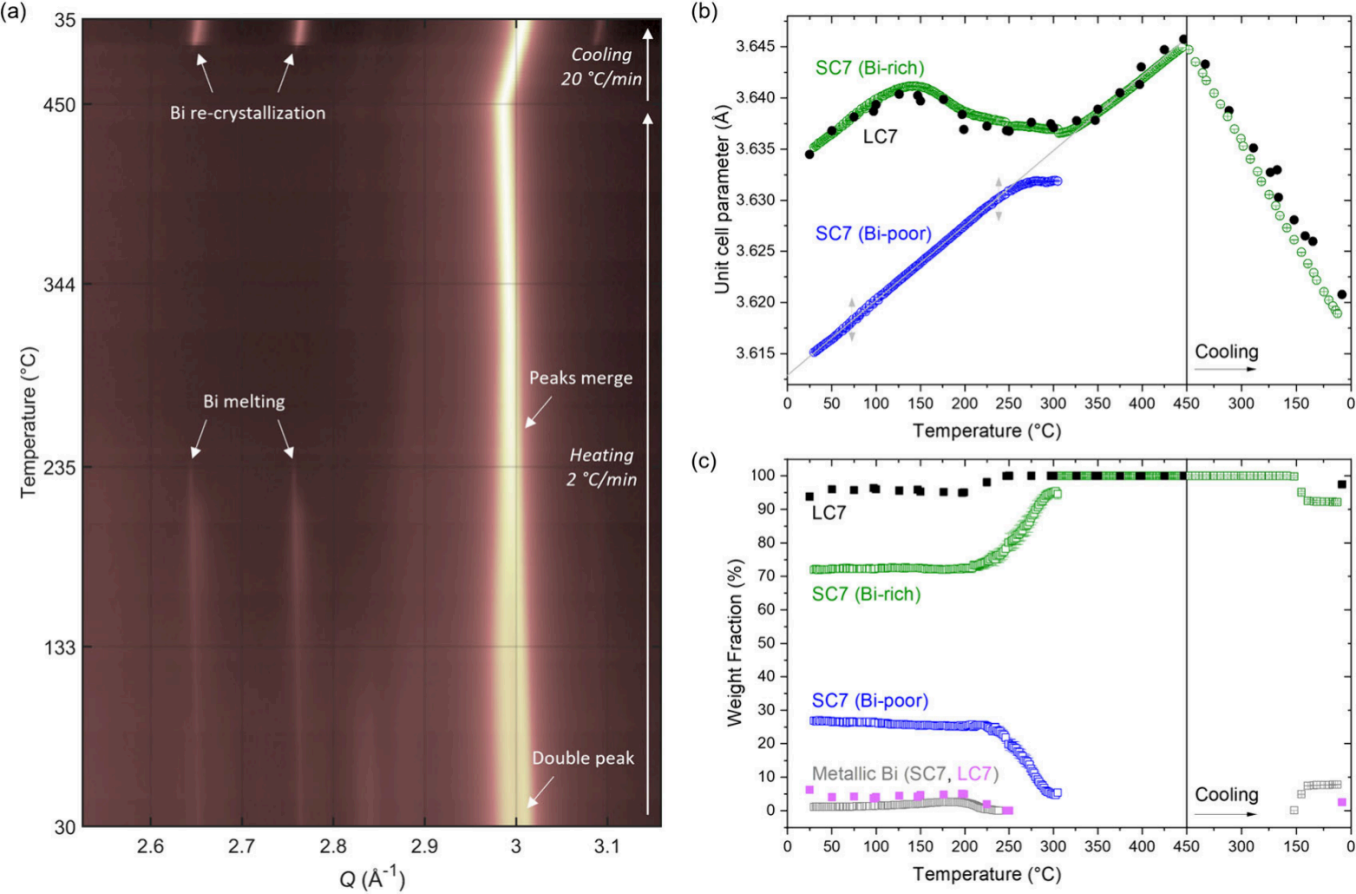
(a) Contour plot of selected *Q*-region of VT SPXRD data collected on the SC7 sample. Full *Q*-range data of SC7 and LC7 samples can be found in Figure S8 and S9, respectively. (b) Refined unit cell a-parameter of the Cu_1–*x*_Bi_*x*_ phase in LC7 (black), and of both Cu_1–*x*_Bi_*x*_ phases of SC7 (Bi rich in green, and Bi poor in blue) as a function of temperature. The linear fit parameters of the SC7 Bi-poor initial thermal expansion (gray line) are given in Table S4. For the equivalent graph of LC7 showing the temperature range RT-1000 °C, see Figure S10. (c) Refined weight fractions as a function of temperature.

At the same time, as shown in Figure 6c, the amount of rhombohedral metallic Bi, which initially accounts for 1.13(7) wt %, increases slightly reaching 2.63(7) wt % at 182 °C. These results indicate that, at approximately 100 °C, Bi starts diffusing out of the Cu lattice in the Bi-rich phase, causing the reduction in unit cell volume of the Cu_1–*x*_Bi_*x*_ phase and the increase in weight fraction of segregated crystalline Bi in rhombohedral *R*-3*m* space group. This takes place up to 300 °C, where no further diffusion of Bi from the Cu_1–*x*_Bi_*x*_ phase takes place, leading to the subsequent linear increase in the unit cell parameter following thermal expansion. Notably, above 182 °C the crystalline Bi phase diffraction peaks start to gradually diminish, completely disappearing at approximately 250 °C, which corresponds to the melting of Bi (Bi melting point = 271.4 °C).^42^

Upon cooling back to RT, the Cu_1–*x*_Bi_*x*_ lattice shrinks, as expected with decreasing temperature. At approximately 150 °C, segregated rhombohedral Bi recrystallizes, reaching 7.8(1) wt % at RT. Once cooled to RT, the unit cell parameter of the single cubic Cu_1–*x*_Bi_*x*_ phase present is 3.618930(7) Å. This parameter is smaller than the initial unit cell of the Bi-rich phase (3.63650(3) Å), but larger than that of the Bi-poor phase (3.61612(2) Å). Moreover, it is 0.12% larger than the unit cell of the pure Cu NWs sample (3.61446(1) Å). This finding indicates that not all Bi has diffused out of the lattice, but a rather a small amount remains present after thermal treatment, and this amount of Bi doping is stable within the probed temperature range.

An equivalent trend in unit cell size with increasing temperature is observed for the LC7 sample, where a single Cu_1–*x*_Bi_*x*_ phase was initially observed with a unit cell parameter similar to that of the Bi-rich SC7 (see black symbols in Figure 6b,c). Not only is the trend the same, but both samples exhibit practically identical values in unit cell parameter as a function of temperature. This result confirms that the diffusion of Bi due to heat is consistent and it takes place at the same temperatures across different Cu_1–*x*_Bi_*x*_ samples. As was observed in the SC7 sample, the diffraction peaks of the metallic Bi initially present in LC7 also disappear at approximately 250 °C. The LC7 sample was heated to 1000 °C, to test the thermal stability to higher temperatures (see Figure S9). The lattice parameter from 450 °C onward follows a linear increase due to thermal expansion (see Figure S10). At 850 °C, the alumina template crystallizes into nanosized domains of γ-alumina (see Figures S9 and S11), which remains crystalline once cooled down to RT. Like for the SC7 sample, in LC7 a small amount of Bi (2.6(1) wt %) recrystallizes when cooled to RT after being heated to 1000 °C. However, it is a lower amount than what was initially present in the as-synthesized LC7 (6.2(3) wt %). The lattice parameter of the Cu_1–*x*_Bi_*x*_ phase of LC7 once cooled to RT is 3.62077(1) Å. This result, once again, corroborates that there is a certain amount of Bi that remains in the NWs after heating, and it is stable up to high temperatures of 1000 °C.

### Variable Temperature (VT) SPXRD with Fast Heating and Quenching

In order to further investigate the thermal stability (specifically whether faster heating and cooling rates influence the diffusion of Bi from the Cu matrix) as well as the local NW structure (see the next section), variable temperature total scattering data were collected on the SC7 NW sample. Here, the sequential collection of total scattering datasets started at ambient conditions with 78 s time resolution. While continuously collecting TS data, the capillary was then rapidly heated to 400 °C, by translating in the hot air blower preheated to the target temperature. After 20 min at 400 °C, the capillary was quenched back to room temperature by retracting the hot air blower. Due to the small sample volume, almost instantaneous heating and quenching were achieved. The target temperature of 400 °C was chosen based on the slow heating experiment, which showed a stable unit cell size at 400 °C, with a linear increase in size due to thermal expansion.

Figure 7a shows a contour plot of the time-resolved synchrotron X-ray TS data (selected *Q*-range) collected on a new batch of sample SC7, denoted here as SC7-B, before, during, and after heating to 400 °C for 20 min. At all three stages, the main crystalline phase observed has a cubic *fcc* structure (space group *Fm*-3*m*), i.e., isostructural to Cu (orange arrows in Figure 7a). As for the original SC7 sample discussed in the RT section, the high angle diffraction peaks of the TS data hint that two Cu_1–*x*_Bi_*x*_ *fcc* phases are present in the sample (i.e., a Bi-rich and a Bi-poor phase). However, due to the high energy of the beam, the two phases cannot be resolved in the TS data as the peak splitting is only slightly visible in the low intensity high Q peaks. Therefore, the sample used for TS experiments, denoted as SC7-B, was modeled with only a single Cu_1–*x*_Bi_*x*_ phase in a cubic *fcc* structure. In addition to the main Cu_1–*x*_Bi_*x*_ phase, a minor amount of crystalline metallic Bi (space group *R*-3*m*) is initially observed to be present (blue arrows). Upon heating the sample to 400 °C, the secondary Bi phase melts (Bi melting point is 271.4 °C),^42^ resulting in the disappearance of the rhombohedral Bi Bragg peaks and emergence of a broad diffuse scattering peak (green arrow). This is consistent with the slow heating VT results, which showed melting of the rhombohedral Bi at around 250 °C. After 20 min of heating at 400 °C, the sample was quenched, leading to recrystallization of a larger amount of metallic rhombohedral Bi than initially present prior to heating, as evident from the emergence of more intense Bi peaks.

**Figure 7:**
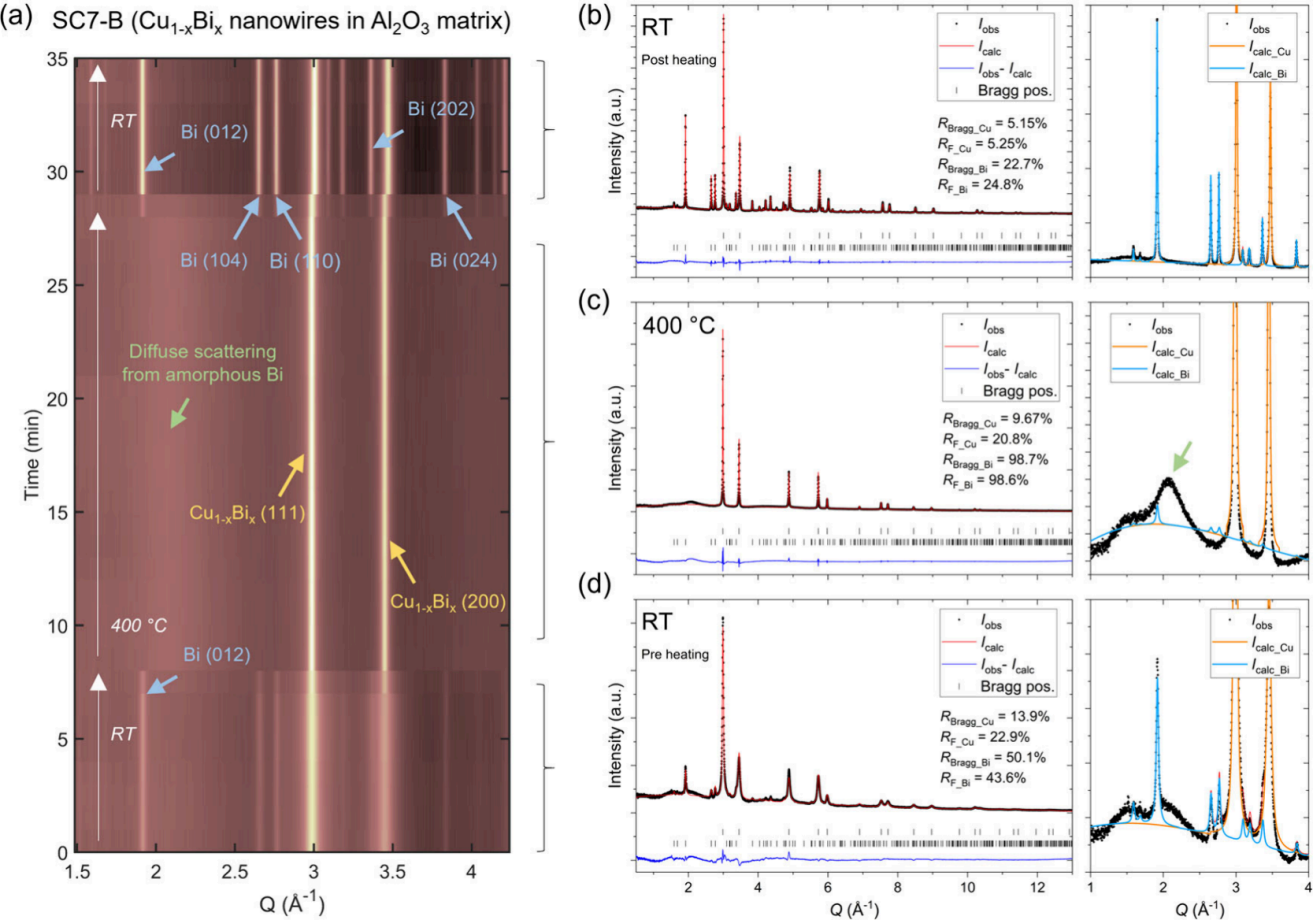
(a) Contour plot of low *Q*-range of time-resolved X-ray TS data collected on the SC7-B Cu_1–*x*_Bi_*x*_ nanowire sample before, during, and after heating to 400 °C. (b–d) Rietveld fits of the Bragg reflections in summed TS data from the three stages. The enhanced *Q*-regions on the right illustrate the disappearance and recrystallization of the secondary Bi phase during the experiment.

Rietveld analysis was carried out for the Bragg scattering in the summed X-ray TS datasets from the three stages (pre, during, and post heating) to extract quantitative compositional, structural, and microstructural information (see Figure 7b–d). Prior to heating, the main cubic Cu_1–*x*_Bi_*x*_ phase observed had a unit cell parameter of 3.6397(1) Å. Notably, Rietveld refinement of equivalent RT TS data collected for the pure Cu NW sample (see Supporting Information) yielded a lattice parameter of 3.61416(8) Å, which is consistent with the one obtained from Rietveld refinements of SPXRD collected at MSPD, ALBA for the same sample (3.61446(1) Å). Consequently, the larger cell parameter of the Cu_1–*x*_Bi_*x*_ sample can be concluded to be consistent with the successful doping of the Cu matrix by the larger Bi atoms. In fact, the unit cell parameter of the Cu_1–*x*_Bi_*x*_ phase in SC7-B is consistent with the unit cell parameter obtained for the Bi-rich phase of the equivalent SC7 sample discussed in previous sections (3.63650(3) Å).

As mentioned earlier, the high symmetry of the cubic Cu_1–*x*_Bi_*x*_ structure, which has only one Wyckoff atomic position, prevents refinement of the respective Cu and Bi occupancies, as they fully correlate with the scale factor, causing fit divergence. Thus, the composition of the main cubic phase must be either inferred/estimated from the unit cell parameter or characterized by complementary techniques. In this case, in the multiphase refinements of all datasets, the atomic structure of the main phase was fixed to the nominal Cu_0.93_Bi_0.07_ composition established by EELS, although the observations indicate that the amount of Bi dopant within the Cu lattice is likely to decrease during/after heating. This was done to avoid overestimation of the Cu_1–*x*_Bi_*x*_ phase weight fractions (due to the higher X-ray scattering power of Bi compared to Cu), which, in turn, would lead to underestimation of the secondary crystalline Bi weight fractions. Thus, in addition to the 7 mol % of Bi in the nanowire lattice, a secondary Bi phase weight fraction of 8.0(2) wt % (equivalent to 3.0(1) mol %) was obtained prior to heating. Upon heating, the Bragg peaks of the Bi phase disappear, while the lattice parameter of the Cu_0.93_Bi_0.07_ phase increases to 3.64154(6) Å. Whether the increase in lattice parameter is solely due to thermal expansions or may have a contribution from incorporation of some of the melted Bi into the structure is not clear from the TS data. However, given the obtained results from the slow heating experiments, where heat leads to extraction of Bi from the Cu lattice, it is safe to assume that the increase in lattice parameter at 400 °C is exclusively due to thermal expansion. While the scale factor parameter for the Bi phase was allowed to refine during the heating step, the resulting minor crystalline Bi weight fraction of 1(1) wt % is undoubtedly an artifact arising due to background correlations (see insert in Figure 7c). Upon quenching, a considerably larger amount of secondary rhombohedral Bi phase (i.e., 19.4(2) wt % or 7.8(9) mol %) than initially present prior to heating (i.e., 8.0(2) wt % or 3.0(1) mol %) is observed to recrystallize. This finding further supports that Bi was successfully incorporated into the original Cu_1–*x*_Bi_*x*_ nanowire structure and that it has diffused outside the Cu lattice due to heating and recrystallized as segregated rhombohedral metallic Bi upon cooling. Considering the additional crystalline Bi formed (7.8(9) mol % – 3.0(1) mol % = 4.8 mol %), this would indicate that approximately 2.2 mol % of the original 7 mol % Bi still remains in the Cu lattice. The refined post heating cell parameter of 3.61887(4) Å also remains slightly larger than the 3.61416(8) Å obtained for the pure Cu nanowires, which is consistent with some Bi remaining in the structure, and with the results obtained in the slow heating/cooling experiment (final unit cell parameter 3.618930(7) Å). Notably, equivalent measurements were conducted on released Cu_1–*x*_Bi_*x*_ nanowires to check whether the alumina template influences the observed Bi diffusion. However, the same results were observed for the embedded and released NWs (see “Released Cu_1–*x*_Bi_*x*_ Nanowires” in Supporting Information).

### Postannealing Morphology and Microstructure of NWs: STEM

To investigate the morphological and microstructural changes occurring in the NWs as a consequence of heat treatment, an *ex situ* annealing experiment (see Methods section) was carried out for the SC7 sample, followed by a microstructural analysis using STEM and EELS. Here, a noticeable change in the NWs’ morphology was readily observed with respect to the as-synthesized NWs. As shown in Figure 8a, the annealed SC7 NWs exhibit wedge-shaped fractures along the length of the NWs, which were not present prior to heating. Small spherical like particles were found next to some of these fractures, as shown in Figure 8b. Interestingly, the HAADF images show narrow, well-delimited bright regions in the NWs (Figure 8c–e), indicating a change in composition between regions exhibiting different contrasts. High resolution STEM imaging of the interface (Figure 8d) shows that both regions are crystalline with apparent atomic coherence between the two structures. Compositional analysis derived from EELS data (Figure 8e) indicates that the bright regions of the NWs correspond to pure Bi, while the darker areas contain both Cu and Bi. Elemental mapping derived from EELS line scan across the thickness of the NW indicate a (2.6 ± 0.7)% Bi content in the core and a higher content on the surface (see Figure 8f,g). As shown in Figure 8e, a thin oxide layer is also present on both the NW surface and at the interfaces between the Bi and Cu_1–*x*_Bi_*x*_ regions, consistent with the formation of an amorphous bismuth oxide layer, as observed on the surface of the as-synthesized NWs. In addition, a few large spherical-like particles were present in the sample, which were confirmed by EELS to be Bi aggregates with a surface oxide layer (Figure 8h). Most likely, these aggregates form due to Bi segregation out of the Cu matrix during annealing.

**Figure 8:**
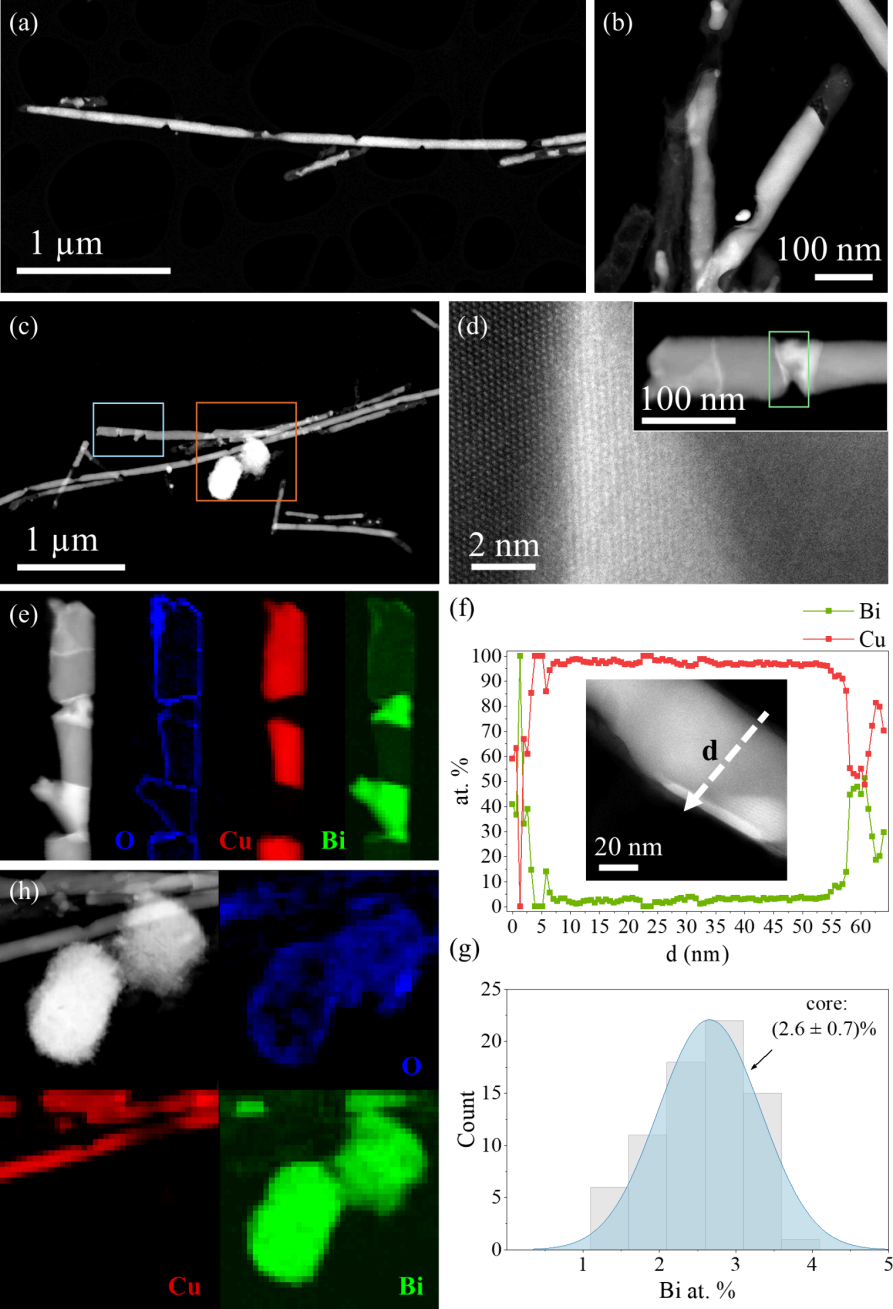
(a–d) HAADF images of postannealed SC7 NWs showing: (a) a NW with wedge-shaped fractures along its length, (b) a fracture in a NW containing a bright spherical particle next to it, (c) low magnification view of NWs with well-defined regions of different contrast, and (d) high resolution STEM image of the interface between a brighter and darker region of the NW shown in (c). (e) HAADF of the NW marked with a blue square in (c) and corresponding spatially resolved EELS maps the O *K*, Cu *L*_2,3_, and Bi *M*_4,5_ edges (in blue, red, and green, respectively). (f) Atomic % composition for Cu and Bi, obtained from EELS line scan across the thickness of the NW (see the white arrow in the HAADF image inset), showing an increase in Bi and reduction of Cu on the surface. (g) Corresponding histogram quantifying the Bi content within the bulk of the NW resulting from data in (f). (h) HAADF image of NWs and spherical particles marked by an orange rectangle in (c), and corresponding EELS maps of oxygen, copper and bismuth. Note that the chemical composition mapping in (e,h) is not normalized and is nonquantitative.

These observations are consistent with the obtained results from the *in situ* heating experiments, where crystallization of segregated rhombohedral Bi was observed in the annealed NWs during the cooling step, as a consequence of a reduction of Bi content in the Cu_1–*x*_Bi_*x*_ phase. Furthermore, the remaining Bi content in the core of the Cu_1–*x*_Bi_*x*_ regions of the NWs obtained by EELS, of approximately 2%, is also consistent with 2.2% extracted from the *in situ* diffraction study, corroborating the robustness of the analysis and reproducibility of the Bi diffusion with temperature.

Combining the *in situ* diffraction and *ex situ* microscopy analyses, we propose a mechanism for Bi diffusion with temperature, following the steps illustrated in Figure 9. During heating, Bi atoms start migrating toward the NWs’ grain boundaries. Upon cooling, Bi recrystallizes at the grain boundaries, leading to a reduction in Bi content inside the NWs, and the emergence of well-defined localized regions of crystalline rhombohedral metallic Bi. In some cases, Bi exsolution takes place in these regions, leaving wedge-shaped fractures and in some cases breaking the NWs. This simultaneously leads to the formation of larger agglomerates of metallic Bi via Ostwald ripening, covered by an amorphous bismuth oxide layer.

**Figure 9:**
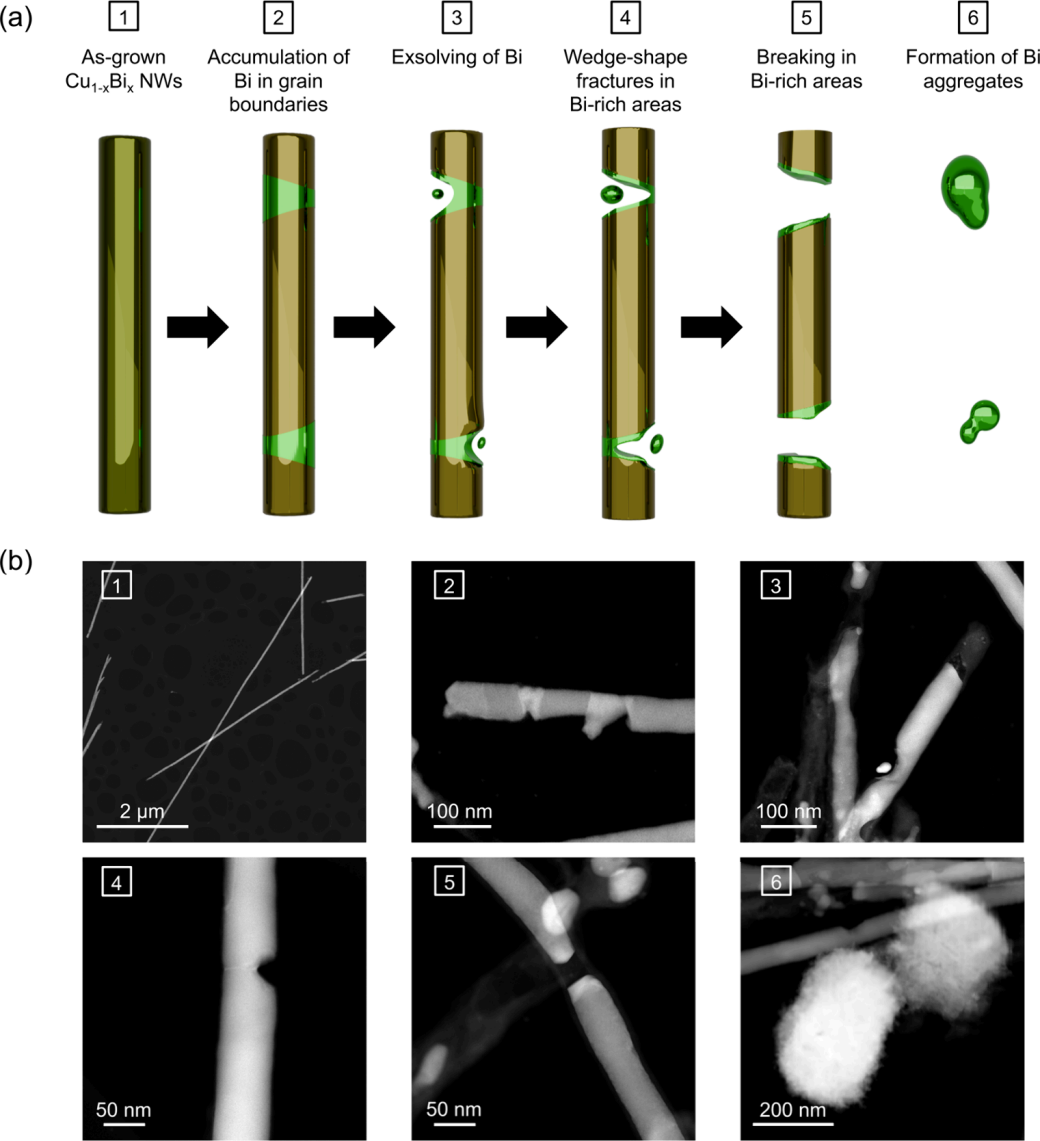
(a) Schematic representation of the mechanism for Bi segregation in Cu_1–*x*_Bi_*x*_ NWs with heating. See the explanation in text. (b) HAADF images showing the events represented schematically in (a).

### Local Atomic Structure: Pair Distribution Function Analysis

Determining the position of Bi in the structure by conventional powder X-ray diffraction (Bragg scattering) analysis is not possible unless the Bi is highly ordered across many unit cells, giving rise to superstructure peaks, none of which were observed here in the high-angular resolution SPXRD data. Given the relatively low amount of Bi within the samples, even in the highly doped ones (7 at. % Bi), it is more likely to be incorporated in a disordered way or with only local order. In TS with PDF analysis, both the Bragg and diffuse scattering signal is utilized, allowing local structural features to be examined.^43^ While the nature of the Bi doping into the Cu lattice is most likely substitutional, i.e. occupying one of the Cu atomic positions in the lattice, it could in principle also be interstitial, i.e., occupying one of the voids in the Cu structure. Notably, given the larger atomic radius of Bi (van der Waals radius = 207 pm) compared to Cu (van der Waals radius = 140 pm), the introduction of interstitial Bi in the lattice would likely give rise to considerable local strain and possibly the formation of Cu vacancies. The nominal Bi content in the sample (7 at. %) is relatively low; however, the higher X-ray scattering power of Bi (*Z* = 83) compared to Cu (*Z* = 29) should make deviations from a disordered cubic lattice (i.e., local Bi ordering) equivalently more visible in the PDF.

To unveil the nature of the Bi doping in the Cu lattice (substitutional vs interstitial, and potential presence of local ordering) both at ambient conditions and during/after heating, the local structure of the NWs was investigated by PDF analysis of the X-ray total scattering data. Figure 10a shows the obtained PDF of the SC7-B sample prior to heating along with simulated PDFs for the structures of Cu, Bi, and Cu with 7% interstitial Bi illustrated in Figure 10b. A list of the low-*r* atomic correlations for the Cu structure is given in Table S5. In the Cu structure, the nearest neighbor Cu–Cu correlation peak is located at *r* = 2.57 Å, which also corresponds to the first peak observed in the experimental PDF from the sample (see Figure 10a). If interstitial Bi was present in the structure (unlikely given its larger atomic radius), the resulting diffuse scattering signal (not taking into account any local lattice distortions/strain or vacancy formations) should give rise to a Cu–Bi correlation at *r* = 1.82 Å. However, no peaks indicating the presence of interstitial Bi are observed in the PDF. Instead, the experimental PDF is in good agreement with the simulated PDF of the cubic Cu structure, indicating incorporation of Bi into the Cu positions in the lattice.

**Figure 10:**
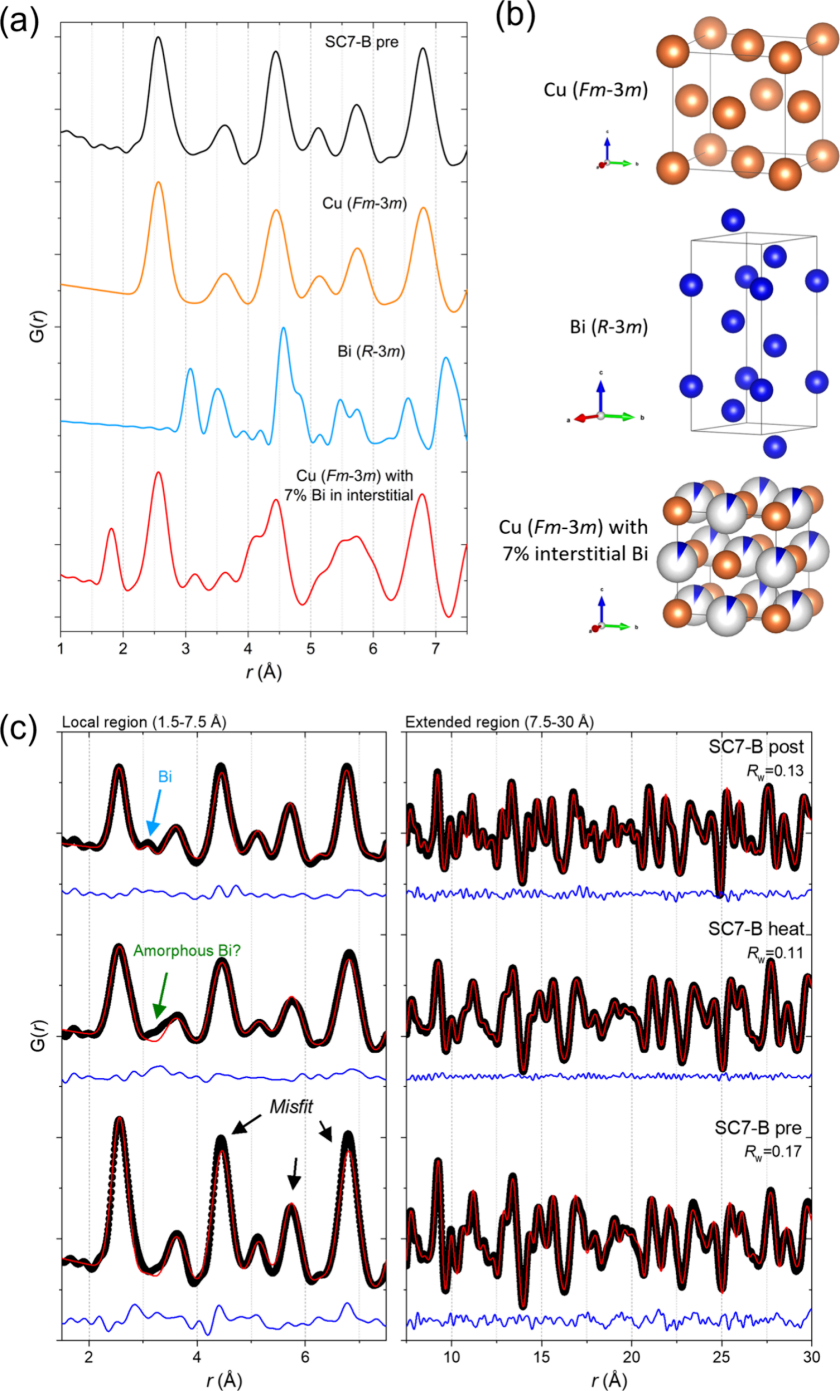
(a) Comparison between collected PDF data of the unreleased Cu_1–*x*_Bi_*x*_ NWs (SC7-B) at ambient conditions (black), and calculated PDFs of pure Cu in space group *Fm*-3*m* (orange), pure Bi in space group *R*-3*m* (blue) and metallic *Fm*-3*m* Cu with 7% interstitial Bi. (b) The atomic structures of Cu, Bi, and Cu with interstitial Bi corresponding to the simulated PDFs. (c) PDF fits for the SC7-B sample collected before (bottom), during (mid), and after (top) heating, all fitted by the same two-phase (Cu_0.93_O_0.07_ and Bi) model.

Figure 10c shows the PDFs and corresponding structural fits for the SC7-B sample before, during, and after heating to 400 °C. The real space structural fit to the PDF before heating is mostly in good agreement with the average crystal structure from the Rietveld analysis of SPXRD data discussed earlier. For the PDF prior to heating, the first two peaks are well fitted, while a misfit can be observed for the peaks at ∼4.46, 5.75, and 6.81 Å (see black arrows). This finding could indicate that rather than Bi being substituted randomly into Cu positions in the lattice, some degree of local Bi ordering exists. At 400 °C, the misfits discussed above disappear as the Bi either disorders or migrates out of the Cu lattice. Instead, a small feature appears at ∼3.3 Å (green arrow), which may be associated with the predominant Bi–Bi bond length in the amorphous melted Bi at high temperature. Post heating, the fit to the main Cu phase remains good indicating that the majority of the Bi has either left the structure or remains disordered. Notably, a peak at ∼3.1 Å associated with the metallic Bi appears after heating.

The observed misfits to the PDF of the Cu_0.93_O_0.07_ NWs prior to heating could indicate that the strongly scattering Bi atoms (compared to Cu) cannot sit next to each other in nearest neighbor (*r* = 2.57 Å) or next-nearest neighboring (*r* = 3.64 Å) sites, but rather preferably order on the sites with interatomic distances of ∼4.46 and 6.81 Å. This idea is illustrated in the expanded (2 × 2 × 2) unit cell shown in Figure 11, where Bi has been placed on a set of specific positions separated by ∼4.46 (black arrows) and 6.81 Å (green arrows). No further noteworthy misfits are observed for higher *r*-values indicating that the Bi ordering is limited to a length scale of <2 unit cells. Thus, to test the proposed Bi ordering, fits to the PDF of preheated SC7-B were carried out using both the disordered and ordered model (including metallic Bi as a secondary phase) limiting the fitting range to 10 Å. As shown in Figure 11, the quality of the fit to the local structure improves when the ordered model is used, with the intensity of the peaks being more accurately described.

**Figure 11:**
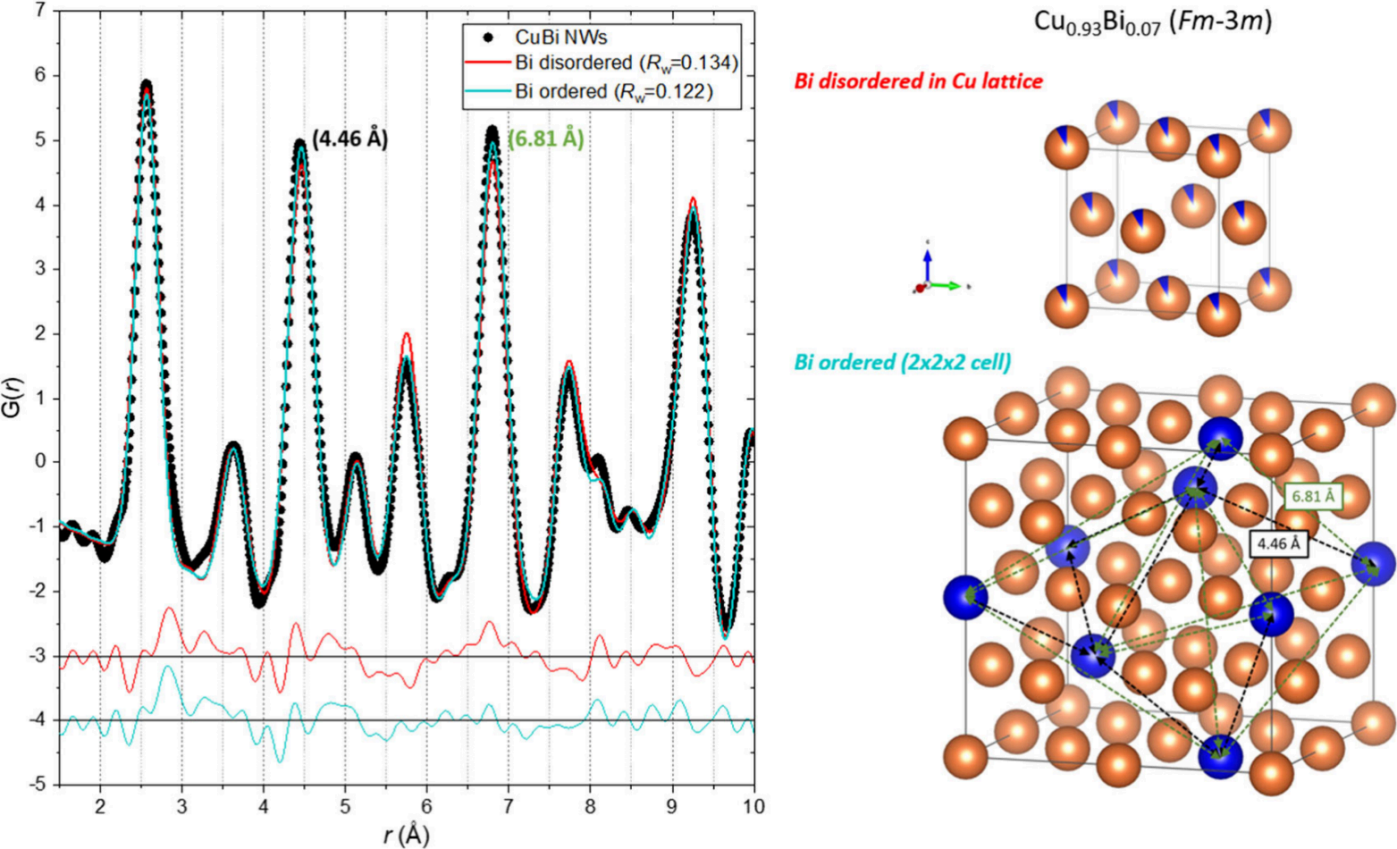
PDF of the preheated SC7-B sample fitted by structural models with disordered (red) and locally ordered (cyan) Bi. The disordered and ordered structures are illustrated on the right. Atomic structures illustrated using the software VESTA.^44^

## Discussion

The present study demonstrates how the composition and microstructure of Bi-doped Cu nanowires can be tuned by varying the synthesis parameters, providing a mechanism to tailor their properties for spintronic applications. In addition, we investigated the effects of slow and fast heating on the crystal structure and microstructure of the NWs, mimicking the expected thermal conditions associated with prolonged and pulsed current operation in spintronic devices, respectively. The intense Joule heating generated at current densities of 10^11^–10^12^ A/m^2^ poses a significant practical challenge for the thermal management and long-term device stability, particularly in nanoscale architectures.

The slow-heating experiments reveal that the Cu_1‑*x*_Bi_*x*_ nanowires remain structurally stable without any detectable phase segregation or degradation up to approximately 140 °C. However, beyond this temperature, the crystal structure becomes progressively affected by the thermal energy, with the lattice exhibiting a transition from positive to negative lattice thermal expansion in the ∼140–300 °C range, which is associated with Bi migrating out of the structure. This suggests that temperatures below 140 °C must be maintained during operation in a spintronic device if structural changes are to be avoided. Upon cooling, the Bi is found to recrystallize, a phenomenon also observed under fast-heating conditions. Our diffraction and microscopy data analyses consistently show that, even after thermal treatment, a residual Bi content of about 2 at. % remains within the nanowires. Although this represents a considerable decrease from the initial 7 at. % Bi level, a pronounced spin Hall effect is still expected, since large negative spin Hall angles (≈ −0.24) have previously been reported for CuBi alloys with much lower Bi concentrations (∼0.5%) as a result of skew scattering from Bi impurities.^6^

The *in situ* SPXRD and *ex situ* microscopy data also indicate the emergence of segregated rhombohedral Bi with temperature, particularly near grain boundaries. The precise impact of such segregation on the spin transport properties remains an open question. Nonetheless, there is growing evidence that nonuniform distributions of heavy-element impurities, especially at interfaces or grain boundaries, can influence spin scattering behavior. Fedorov et al. proposed that Bi clustering and interface roughness may help explain discrepancies between theoretical and experimental SHA in CuBi alloys.^9^ Similarly, Tatsuoka et al. recently demonstrated that spin–orbit torque (SOT) efficiencies in CuBi depend sensitively on the Bi composition and device structure, likely due to variations in local spin–orbit interaction and spin backflow suppression.^11^

While the role of segregated Bi at grain boundaries is not fully established, the presence of high-Z atoms in such positions could feasibly enhance skew scattering contributions to the SHE, as theorized by Fert and Levy in their resonant scattering model.^45^ Consequently, further systematic studies, specifically involving local spin transport measurements on CuBi systems with independently varied Bi concentration and crystalline quality, will be essential to disentangle the respective contributions of composition and microstructure to spin transport behavior and to assess whether Bi segregation at grain boundaries enhances or hinders the spin Hall effect. The present findings establish the importance of understanding the structural characteristics and controlling the thermal conditions in Cu_1–*x*_Bi_*x*_ NWs, and provide the framework to study spin transport anomalies in CuBi systems with varying composition and structure.

## Conclusions

This study provides a comprehensive investigation of the microstructure, structure, and thermal stability of Bi-doped Cu nanowires synthesized using template-assisted electrodeposition. Macroscopically averaged X-ray diffraction techniques were combined with local electron microscopy measurements for this aim. The structural analysis reveals that by varying the concentration of tartaric acid (TA) in the electrolyte solution during the template-assisted electrodeposition, control over crystalline domain sizes and Bi distribution is achieved. 4D-STEM analysis shows that smaller crystalline domains (∼200 nm) with Bi accumulation at the grain boundaries are achieved at lower TA concentrations. In contrast, higher TA concentrations promote formation of larger crystalline domains (> 500 nm) and uniform Bi incorporation into the Cu lattice. This is evidenced by EEL spectra and a linear increase in refined lattice parameter with Bi doping, consistent with Vegard’s law. To investigate the thermal stability of the NWs, variable temperature SPXRD data were collected at 450 and 1000 °C, with slow heating (2 °C/min) and cooling (∼20 °C/min) rates. Rietveld analysis of the data reveals that, above 100 °C, Bi begins diffusing out of the Cu lattice, with significant migration occurring between 140–350 °C. In addition, a small amount of metallic Bi (1–8 wt %), which melts at approximately 250 °C, is often present in the as-synthesized samples. When the samples are cooled back down to RT, metallic Bi (both initially present and diffused from the Cu matrix) recrystallizes. Importantly, both for slow and fast heating/cooling experiments, including those to 1000 °C, a fraction of Bi remains present in the Cu lattice once cooled back to RT, as evidenced by the values of the unit cell parameter of the post heated samples being consistently larger than that of pure Cu NWs. Results from all VT experiments indicate that the amount of Bi remaining in the Cu_1–*x*_Bi_*x*_ lattice corresponds to approximately 1–2 at. %. These results are consistent with EELS data of annealed NWs, which show ∼2 at. % Bi in NWs post heating. The heating leads to a change in microstructure with accumulation of crystalline rhombohedral Bi at the grain boundaries, formation of wedge-shaped fractures in the NWs and exsolution of Bi particles. PDF analysis of the TS data was carried out to examine the nature of the Bi integration in the Cu lattice. An improved fit to the PDF was obtained using a model with locally ordered Bi, which supports the hypothesis that Bi atoms in the Cu NWs exhibit a preference for specific interatomic distances rather than random distribution, with a tendency to avoid nearest- and next-nearest neighboring positions. Our findings highlight the intricate relationship between synthesis parameters, structural characteristics, and thermal stability in this nanophase system. This study underscores the importance of tuning synthesis parameters to achieve targeted microstructural properties and thermal behavior, paving the way for the development of high-performance Cu_1–*x*_Bi_*x*_ nanowires tailored for next-generation spintronic devices. Elucidating the structural and thermal behavior of these materials is paramount for optimizing and tuning the device performance under operational heating conditions. Nanowires with smaller crystalline domains, where Bi localizes at grain boundaries, may enhance or diminish spin scattering mechanisms critical for SHE. Further studies into the spin properties of the different NWs are needed to elucidate the structure–property correlation and thus understand and optimize the yet unknown mechanisms driving the giant SHE in CuBi alloys.

## Methods

### Synthesis of Cu_1–x_Bi_*x*_ Nanowires

Cu_1–*x*_Bi_*x*_ NWs (*x* = 0, 0.02, 0.04, 0.07) were synthesized by template-assisted electrochemical deposition in the pores of anodized aluminum oxide (AAO) templates with pore sizes of 50 nm and thicknesses around 40 μm, which were prepared by anodization onto high-purity (99.999%) Al discs of 25 mm in diameter. The anodization was conducted for 5 h in a two-electrode electrochemical cell at room temperature, using a 0.3 M oxalic acid (C_2_H_2_O_4_) solution under continuous stirring. To maintain a constant temperature during anodization, the cell was placed on a copper plate equipped with a cooling circuit, through which a refrigerating liquid was circulated and connected to a cryothermostat (see Figure S14). The applied voltage was maintained at a constant 40 V with the aid of an adjustable DC power supply (EA Elektro-Automatik, Viersen, Germany). A Pt mesh was employed as counter electrode. Following anodization, the residual aluminum layer was chemically etched using a solution of 0.74 M CuCl_2_ and 3.25 M HCl, while an outer aluminum ring was preserved to provide mechanical support and facilitate handling in the following steps. Lastly, the pores were opened using a 5% (v) H_3_PO_4_ at room temperature for 1.5 h. The complete anodization procedure has been described in detail in a previous publication from our group.^18^ Prior to electrodeposition, one side of the nanoporous templates was coated with a Ti­(15 nm)/Au­(150 nm) thin layer deposited by sputtering using a Leica EM ACE600 sputter coater. This conducting layer acts as a working electrode for electrodeposition.

The electrolyte was prepared by mixing varying amounts (0.14, 0.29, or 0.58 g) of Bi­(NO_3_)_3_·5H_2_O, 1.5 g of CuSO_4_·5H_2_O, 17.43 g of KNO_3_ and varying amounts (7.44, 14.88, 22.32 g) of Tartaric acid (TA) (C_4_H_6_O_6_) in 150 mL of a solution of 10% vol. glycerol in deionized water. This led to electrolyte solutions with final Bi­(NO_3_)_3_·5H_2_O concentrations of 2, 4, and 8 mM, and different amounts of TA. The Bi­(NO_3_)_3_·5H_2_O concentration was varied in order to obtain different compositions of the prepared Cu_1–*x*_Bi_*x*_ NWs, while the TA was added to examine its role as a chelating agent, specifically to study its impact on Bi solubility and crystallite sizes. HNO_3_ was subsequently added to the mixture, under magnetic stirring, to lower the pH until complete dissolution of the electrolyte salts was achieved at around pH = 0.9. The electrodeposition was carried out at room temperature using a three-electrode electrochemical cell with a Pt mesh as the counter electrode and an Ag/AgCl (3 M NaCl) electrode as the reference electrode. The growth of the NWs was controlled using a Metrohm-Autolab PGSTAT potentiostat with an applied growth potential of −0.1 V during 10 s for nucleation and −0.05 V during the rest of the growth time. Note that both potentials were referred to the reference electrode. After the synthesis, the Ti­(15 nm)/Au­(150 nm) contact layer was removed by chemical etching using Hg and the NWs were released from the AAO template using a 0.4 M H_3_PO_4_ and 0.2 M H_2_CrO_4_ solution, followed by repeated rinsing with milli-Q water. Once released, the NWs were stored in pure ethanol to avoid oxidation. Note that a vortex mixer rather than sonication was used for the dissolution of the AAO template and subsequent rinsing to prevent the NWs from heating.

### Electron Microscopy

#### STEM and EELS Mapping

STEM-EELS characterization was carried out using a JEOL JEM-ARM200cF aberration corrected electron microscope operated at 200 kV, equipped with a cold field emission gun and a Gatan Quantum spectrometer. For spectrum imaging, the electron beam was scanned along the region of interest, and EEL spectra were acquired with an energy dispersion of 1 eV/channel. Random noise was removed from the EELS data using principal-component analysis.^46^ The background signal was subtracted using a power law fit before integration of the signal in order to produce EELS maps.

#### Four-Dimensional STEM Nanodiffraction Data Collection and Analysis for Crystal-Phase Mapping

4D-STEM nanodiffraction data were collected on a JEOL MonoNEOARM 200F instrument equipped with a monochromator, an imaging aberration corrector and a probe aberration corrector, a Gatan Imaging Filter Continuum HR electron energy-loss spectrometer (EELS) and an energy dispersive X-ray spectrometer (EDS). Data were acquired using a Gatan Rio Camera while operating at 200 keV with a convergence angle of 3.4 mrad and a camera length of 2.5 cm. The py4DSTEM package was used for the data calibrations and crystal-orientation mapping analysis.^26^ Calibrations include correcting shifts of the diffraction pattern, calibrating the rotational offset between the real and the diffraction space, and calibrating the pixel sizes. After the calibrations were performed, the detection of the Bragg peaks from each of the data points was carried out to obtain Bragg vector maps. Then, the average reciprocal lattice vectors were extracted and indexed.

#### Lamella and EDS Mapping

LC7* NWs stored in an ethanol solution were dispersed onto a Si substrate. To protect them from ion damage during the lamella preparation by focus-ion beam (FIB), they were coated by sputtering with a 50 nm thick Au layer using a Leica sputter coater, EM ACE600. Then FEI Versa 3D FIB-SEM was used to prepare the cross-sectional sample, and the above-mentioned JEOL MonoNEOARM 200F microscope was used for the lamella STEM imaging and EDS acquisition. The STEM observations were performed immediately after the preparation of the lamella via FIB in a coordinated manner to prevent oxidation of the NWs cross-section.

#### Annealing of SC7 Sample for Microstructural Analysis

A portion of the alumina template containing the as-synthesized SC7 NWs was placed in an alumina crucible and heated in a CARBOLITE RHF 15/3 furnace to 450 °C at 2 °C/min, following the same heating profile as for the *in situ* SPXRD slow-heating thermal study. Once at 450 °C, the furnace was turned off and allowed to cool down. The NWs were then released from the alumina template using the same procedure as described earlier, and dispersed onto a Holey Carbon-Cu grid.

### Powder Diffraction and Total Scattering Experiments

#### Room Temperature SPXRD

The as-synthesized samples (amorphous AAO template containing the NWs) were crushed into a powder and packed into 0.3 mm Quartz capillaries, which were sealed using two-component epoxy glue. Room temperature (RT) high-angular resolution synchrotron powder X-ray diffraction (SPXRD) data were collected at the BL04-MSPD beamline at the ALBA Synchrotron with an energy of 30 keV,^47^ and at beamline ID22 at the European Synchrotron Radiation Facility (ESRF),^48^ with an energy of 35 keV.^49^ The X-ray wavelengths were calibrated to 0.413560 Å (BL04-MSPD, 30 keV) and 0.354324 Å (ID22, 35 keV), respectively, from Rietveld refinement of powder X-ray diffraction data collected on a NIST LaB_6_ 660b standard.^50^ The capillaries were continuously spun during data collection to improve powder averaging. At BL04-MSPD, ALBA, the diffraction data were collected using the high-throughput position sensitive detector MYTHEN, while at ID22, ESRF, the SPXRD data were collected using the 13-channel Si 111 multianalyzer stage and Dectris Eiger2 × 2M-W CdTe pixel detector.

#### Variable Temperature SPXRD

Variable temperature (VT) SPXRD experiments with slow heating (2 °C/min) and cooling (20 °C/min) between RT and 450 °C or 1000 °C were also carried out on selected samples at the BL04-MSPD beamline at the ALBA Synchrotron and at the ID22 beamlinne at ESRF, using the same conditions as for the RT measurements. The samples were heated using a FMB Oxford hot air blower at ALBA, and a Cyberstar hot air blower at ESRF, mounted on top or underneath the sample. The data were continuously collected with a 1 min and 2.5 min time-resolution at ALBA and ESRF, respectively.

#### Variable Temperature Total Scattering Experiments

Variable temperature X-ray total scattering (TS) experiments with instant heating to 400 °C, holding, and quenching to RT were conducted using the high-resolution powder diffractometer at beamline ID22 at the European Synchrotron Radiation Facility (ESRF).^49^ The X-ray wavelength was calibrated to 0.206689(1) Å (60 keV) from Rietveld refinement of powder X-ray diffraction data collected on a NIST standard LaB_6_ 660b standard.^50^ Data were collected on the NWs still embedded in the alumina template (as for the RT measurements) as well as on released NWs. Both types of samples were packed into Quartz capillaries and sealed using two-component epoxy glue. Data were also collected under ambient conditions for an empty capillary and a capillary containing an empty alumina template. During measurements, the capillaries were spun at 919 rpm and continuously rocked ±1 mm to improve powder averaging. The samples were heated using a retractable Cyberstar hot air blower (RT-400 °C) mounted underneath the sample. The hot air blower was preheated and translated in/out to achieve almost instantaneous heating and quenching of the samples. The scattering data were continuously collected with a 78 s time-resolution (sum of 25 × 1 s exposures + detector readout) using the PerkinElmer 2D detector positioned at a distance of 380 mm behind the sample capillary giving a *Q*_max_ of approximately 25 Å^–1^.

#### Structural Analysis

Rietveld analysis of the Bragg scattering in the diffraction and TS data was performed using the *FullProf Suite* software package.^51^ Fourier transformation of the X-ray total-scattering (Bragg and diffuse sample scattering) data into real-space PDFs was carried out using *PDFgetX3*,^52^ and the real-space structural refinements of the PDFs were carried out using the *PDFgui* software.^53^ The background intensity from a 0.3 mm quartz capillary containing an unfilled alumina template was subtracted from the TS data. The experimental *Q*_damp_ (instrumental damping of PDF peak intensities) was determined to be 0.009 Å^–1^ by fitting of NIST LaB_6_ 660b calibrant PDFs collected in the same instrumental configuration.

## Supplementary Material



## Notes

Tables with overview of synthesized samples and refined structural and compositional parameters; SEM micrograph of a cross section of an alumina template with grown Cu_1–*x*_Bi_*x*_ NWs; additional HAADF-STEM images of a lamella cross-section view of several LC7* NWs; representation of crystal structures; additional Rietveld refinement fits of different samples; comparison of refined weight fractions of the Bi-rich Cu_1–*x*_Bi_*x*_ phase refined as pure Cu vs refined containing 7% Bi; details on theoretical simulation of Joule heating in a Cu NW using COMSOL Multiphysics; contour plots of variable temperature SPXRD data of SC7 and LC7 samples; linear fit parameters of Figure 6a; refined unit cell parameters of LC7 as a function of temperature; contour plots, Rietveld refinements and additional information on released Cu_1–*x*_Bi_*x*_ NWs; list of low-*r* atomic correlations for the Cu *fcc* structure giving rise to peaks in the PDF data (PDF); electrodeposition setup
