# Supplementary material for: Correlating Synthesis, Structure, and Thermal Stability of CuBi Nanowires for Spintronic Applications by Electron Microscopy and in Situ Scattering Methods
Source: ACS Nano. 2025 Dec 2;19(49):41509–27. doi: 10.1021/acsnano.5c09560 (PMC12713782; doi:10.1021/acsnano.5c09560)
Supplement: Supplementary file 1 [file nn5c09560_si_001.pdf]

Electronic Supporting Information for:

Correlating synthesis, structure and thermal stability of CuBi nanowires for spintronic applications by electron microscopy and *in situ* scattering methods

Alejandra Guedeja-Marrón,[1,2] Henrik L. Andersen,[3] Gabriel Sánchez-Santolino,[1,2] Lunjie Zeng,[4] Alok Ranjan,[4] Inés García-Manuz,[5,6] François Fauth,[7] Catherine Dejoie,[8] Eva Olsson,[4] Paolo Perna,[5] Maria Varela,[1,2] Lucas Pérez[1,5] and Matilde Saura-Múzquiz[1*]

[1]*Departamento de Física de Materiales, Facultad de Ciencias Físicas, Universidad Complutense de Madrid, Madrid 28040, Spain*
[2]*Instituto Pluridisciplinar, Universidad Complutense de Madrid, Madrid 28040, Spain*
[3]*Instituto de Ciencia de Materiales de Madrid (ICMM), CSIC, Madrid 28049, Spain*
[4]*Department of Physics, Chalmers University of Technology, Gothenburg 41296, Sweden*
[5]*IMDEA Nanociencia, Madrid 28059, Spain*
[6]*Departamento de Física de la Materia Condensada (IFIMAC), Universidad Autónoma de Madrid, 28049 Madrid, Spain*
[7]*CELLS-ALBA Synchrotron, Barcelona 08290, Spain*
[8]*European Synchrotron Radiation Facility (ESRF), Grenoble 38000, France*

*Corresponding author: matsaura@ucm.es

Table S1. Overview of synthesized samples including sample name, concentration of $Bi(NO_3)_3$ and TA, and electrodeposition potential. Samples with an asterisk, with a more pronounced difference in TA concentration, were used for 4D-STEM measurements.

Sample name	$Bi(NO_3)_3$ concentration (mM)	TA concentration (M)	Electrodeposition potential (V)
SC2	2	0.33	-0.05
SC4	4	0.33	-0.05
SC7	8	0.33	-0.05
LC2	2	0.99	-0.05
LC4	4	0.99	-0.05
LC7	8	0.99	-0.05
LC7*	8	1.32	-0.05
Cu	NA	0.33	-0.3
Bi	8	0.33	-0.07

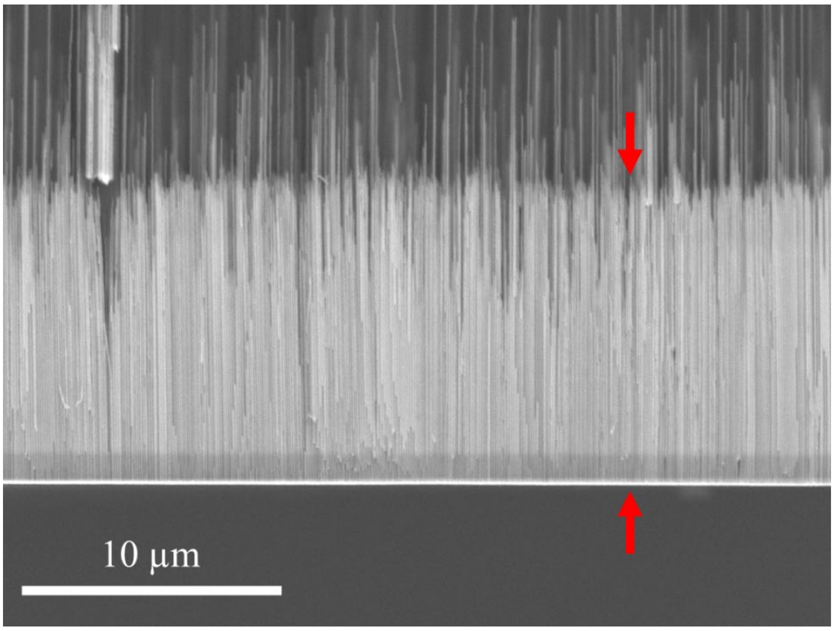

Figure S1: SEM cross-sectional view of an AAO with the embedded $Cu_{1-x}Bi_x$ NWs. The red arrows indicate the total length of the NWs, extended from the conductive layer composed of Ti (10 nm) and Au (150 nm), which appears with a brighter horizontal contrast.

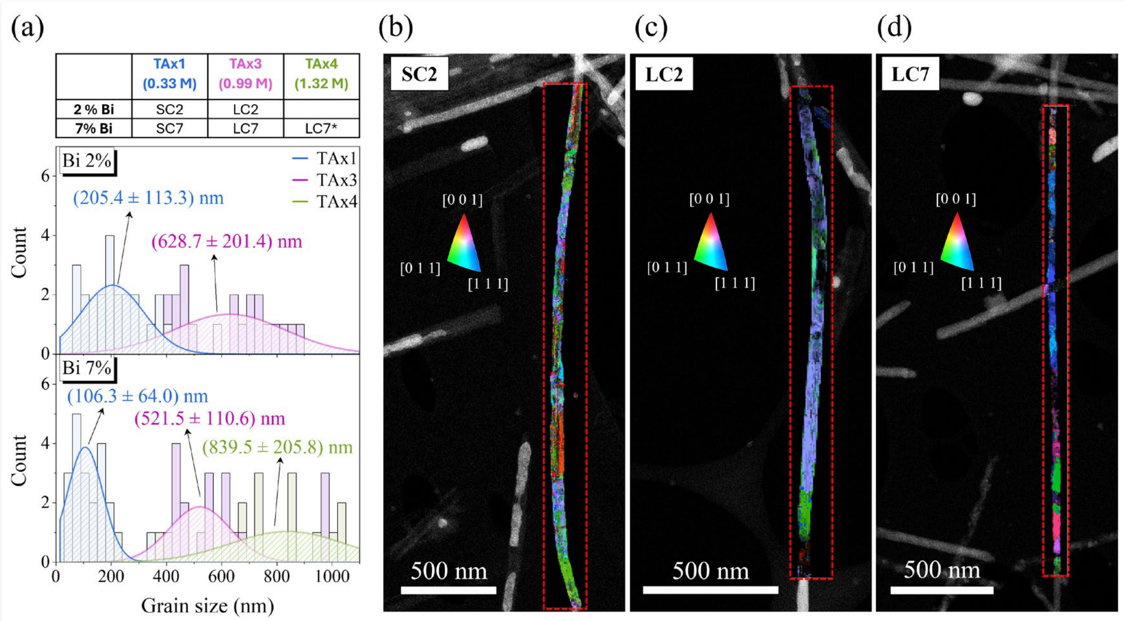

Figure S2: Grain size distribution and crystallographic orientation mapping of $Cu_{1-x}Bi_x$ nanowires synthesized with different tartaric acid concentrations and Bi content. (a) Histograms of grain size distributions obtained from 4D-STEM orientation maps of nanowires prepared with TAx1 (blue), TAx3 (magenta), and TAx4 (green), and table indicating the correlation of TA concentration, Bi content and sample name. The top histogram corresponds to samples with a Bi content of 2% and the bottom one, to samples with 7% Bi. (b-d) Representative 4D-STEM orientation maps of SC2, LC2 and LC7 simples, respectively, along with the color-coded crystallographic orientation legend used for the mapping. The analyzed region is marked by a red dashed rectangle.

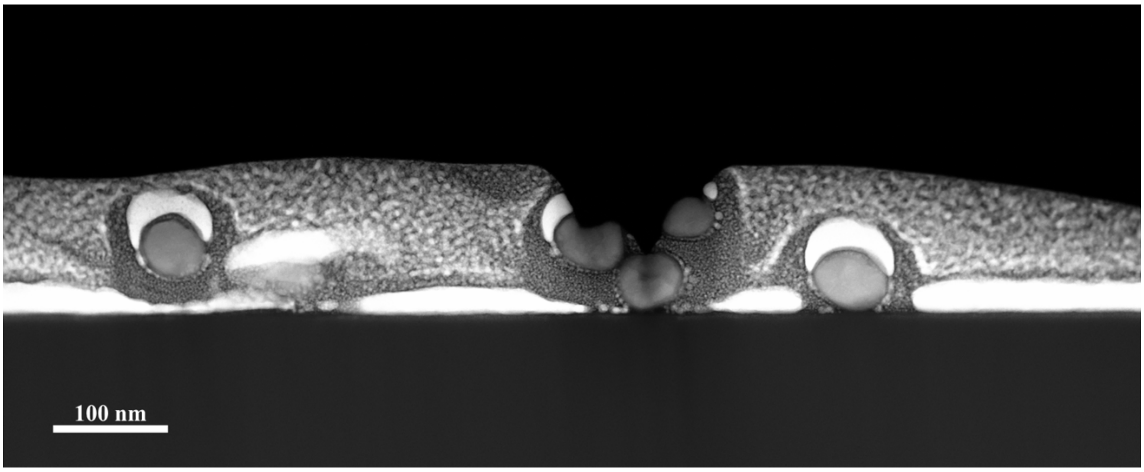

Figure S3: Low magnification HAADF-STEM lamella cross-section view of several LC7* NWs prepared by FIB. For the lamella preparation, the NWs were dispersed onto a Si substrate. Then they were coated with a protective Au layer which can be noticeable in the HAADF-STEM image with a brighter contrast. The lamella trenches were dug in an area where multiple NWs were observed aligned in parallel.

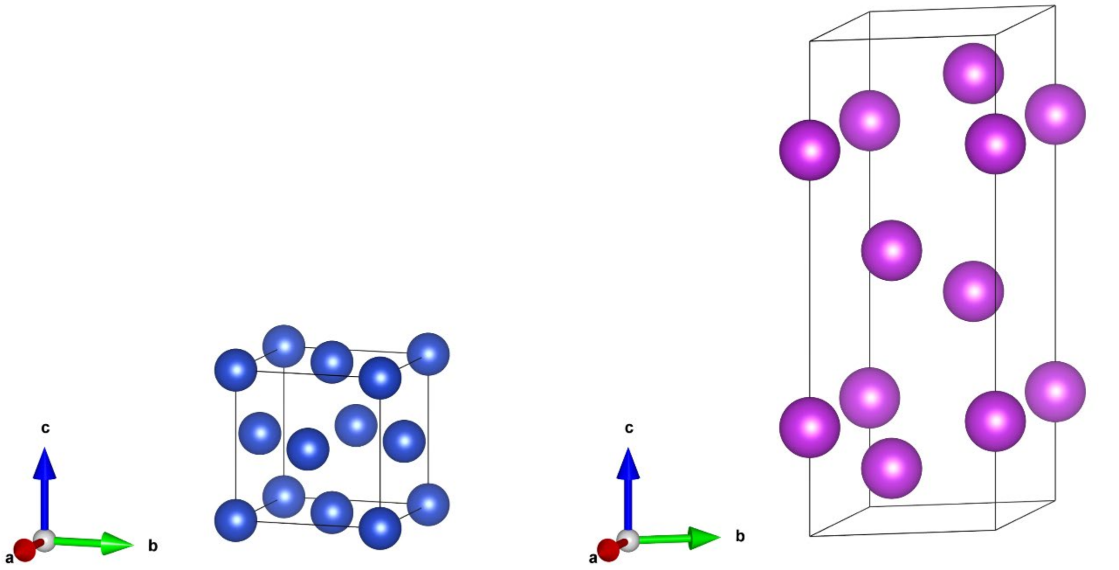

Figure S4. Illustrations of one unit cell of metallic Cu (left) and metallic Bi (right) in cubic *Fm*-3*m* and rhombohedral *R*-3*m*, respectively. Structures represented using VESTA (*J. Appl. Cryst.* (2011). **44**, 1272-1276).

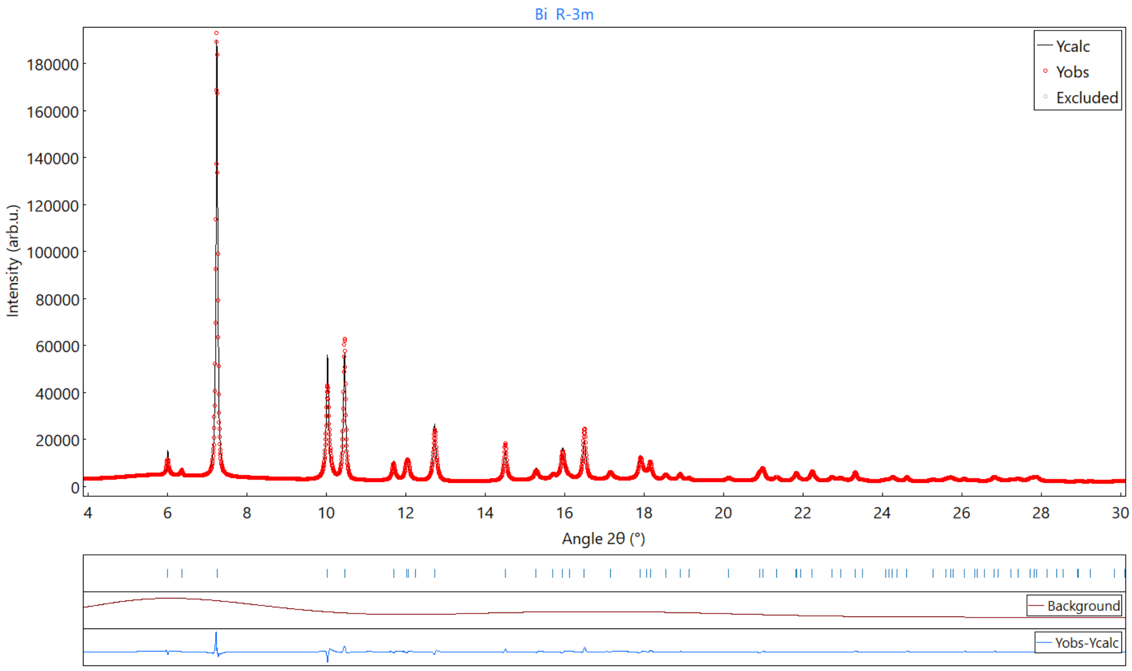

Figure S5: Rietveld refinement of SPXRD data collected on pure Bi nanowires grown by electrodeposition, using the same template as for the studied samples. The data is refined in space group *R*-3*m*.

Table S2: Refined weight fractions of sample SC7, with the Bi-rich $Cu_{1-x}Bi_x$ phase (larger lattice parameter) refined as pure Cu *vs.* refined containing 7% Bi. In both cases, the Bi-poor $Cu_{1-x}Bi_x$ phase (smaller lattice parameter) was refined as pure Cu.

**Refinement model of Bi-rich phase**	**$Cu_{1-x}Bi_x$ Bi-poor (wt.%)**	**$Cu_{1-x}Bi_x$ Bi-rich (wt.%)**	**Metallic Bi (wt.%)**
Refined as Cu	27.8(3)	70.4(5)	1.75(8)
Refined as $Cu_{0.093}Bi_{0.07}$	29.7(3)	68.4(5)	1.88(9)

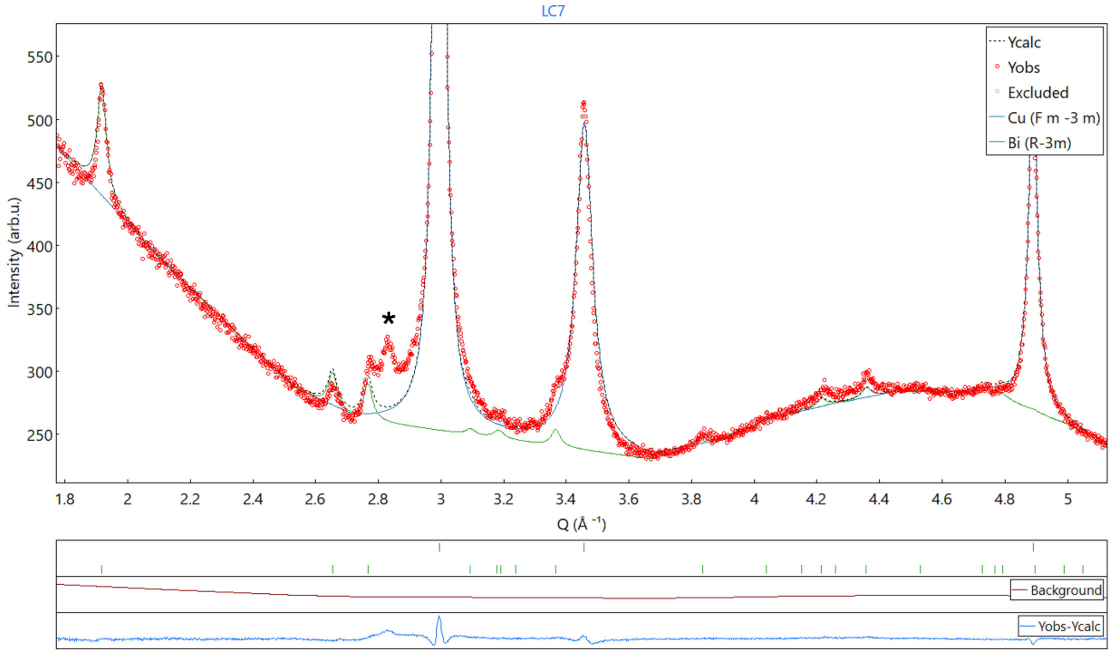

Figure S6: Close up of the Rietveld refinement of SPXRD data collected on sample LC7, showing the experimental data as red dots, and the contribution of the metallic Bi to the refinement as a green line. The refined $Cu_{1-x}Bi_x$ phase (refined as Cu), is shown in blue, and the total refined model is shown as a black dotted line. An additional peak marked with an asterisk is observed, which corresponds to an unidentified impurity.

Table S3: Refined weight fractions, lattice parameters and unit cell volumes of all phases in all samples. For SC7 and LC7, where two $Cu_{1-x}Bi_x$ phases are present, in addition to the refined parameters for each phase, the calculated weighted average (WA) parameters of both $Cu_{1-x}Bi_x$ phases are given.

Sample	Phase	wt.%	*a*-axis (Å)	*c*-axis (Å)	UC volume (Å^3)
**Cu**	Cu	100.0(2)	3.61446(1)	-	47.2205(4)
**Bi**	Bi	100.0(4)	4.54464(3)	11.8661(1)	212.245(3)
**SC2**	$Cu_{1-x}Bi_x$	100.0(3)	3.610010(7)	3.62468(1)	47.2375(2)
**SC4**	$Cu_{1-x}Bi_x$	100.0(4)	3.615389(9)	-	47.2565(4)
**SC7**	$Cu_{1-x}Bi_x$ (Bi-poor)	27.8(3)	3.61612(2)	-	47.2856(7)
	$Cu_{1-x}Bi_x$ (Bi-rich)	70.4(5)	3.63650(3)	-	48.090(1)
	WA $Cu_{1-x}Bi_x$	98.3(3)	3.63073(2)	-	47.8619(9)
	Bi	1.75(8)	4.5440(5)	11.874(3)	212.32(6)
**LC2**	$Cu_{1-x}Bi_x$ (Bi-poor)	66.9(4)	3.61108(1)	3.62295(3)	47.2429(5)
	$Cu_{1-x}Bi_x$ (Bi-rich)	33.1(4)	3.63640(4)	-	48.086(2)
	WA $Cu_{1-x}Bi_x$	100.0(3)	3.61946(2)	3.62740(2)	47.5208(5)
**LC4**	$Cu_{1-x}Bi_x$	93.2(5)	3.63036(2)	-	47.8464(8)
	Bi	6.8(2)	4.5194(8)	11.955(4)	211.46(9)
**LC7**	$Cu_{1-x}Bi_x$	93.8(6)	3.63448(2)	-	48.010(1)
	Bi	6.2(3)	4.519(1)	11.921(6)	210.9(1)

COMSOL simulation of Cu NW

The modeled nanowire has a diameter of 50 nm and a length of 10 µm and is electrically contacted to gold (Au) pads and suspended on a silicon nitride (SiN) substrate. These structural and boundary conditions were implemented to accurately capture the effects of thermal diffusion: three-dimensionally through the nanowire, laterally through both the contacts and substrate, and outward to the surrounding air environment (see Figure S7(a)). The simulation considered induced current densities in the range of 10^{10}–10^{11} A/m^2, with pulse durations varying from few minutes to 1 hour. These conditions are consistent with those typically required for OHE detection. This configuration also reflects geometries commonly used in experimental techniques such as X-ray magnetic circular dichroism (XMCD).

In Figure S7(b), the contour plot illustrates the maximum temperature reached as a function of the applied discrete current densities (in the range of 10^{10} to 10^{11} A/m²) over a total driving time of 60 minutes. The color gradient represents the temperature increase due to Joule heating, starting from room temperature and reaching up to 654 °C. As a reference, the times at which the 250 °C threshold is reached for each current density are indicated. This threshold corresponds to the onset temperature at which phase changes are observed in the system. According to the simulation results, for techniques requiring more than one hour of continuous biasing, it is safe to operate in a stationary regime, i.e., without inducing structural or compositional changes, at current densities below 4.5 $\times 10^{11}$ A/m^2. In contrast, current densities above 6.3 $\times 10^{11}$ A/m^2 are only compatible with techniques involving measurement times shorter than ~15 minutes. Based on the observed trend, it can be inferred that at even higher current densities, only methods with acquisition times on the order of seconds to a few minutes would remain within the thermally stable regime.

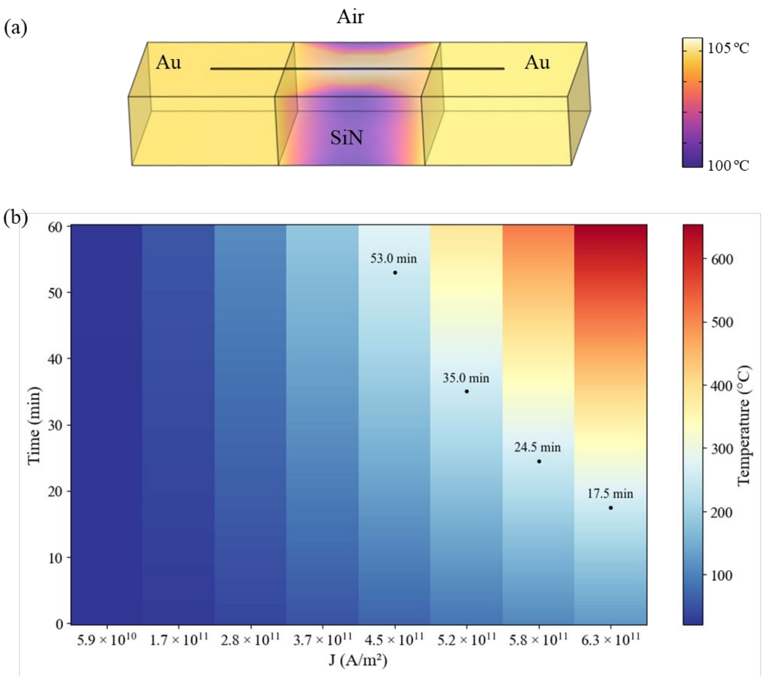

Figure S7: (a) Schematic of the modeled nanowire system (50 nm diameter, 10 µm length) suspended on a SiN substrate and contacted by Au pads. The setup captures 3D thermal diffusion through the nanowire, contacts, substrate, and into surrounding air. (b) Contour plot showing the maximum temperature as a function of applied current density (in units of A/m²) and driving time (up to 60 minutes). The color scale represents temperature elevation due to Joule heating, ranging from room temperature to a maximum of 654 °C. Black dots indicate the times at which the temperature reaches 250 °C for each current density—this threshold marks the onset of phase transitions in the system.

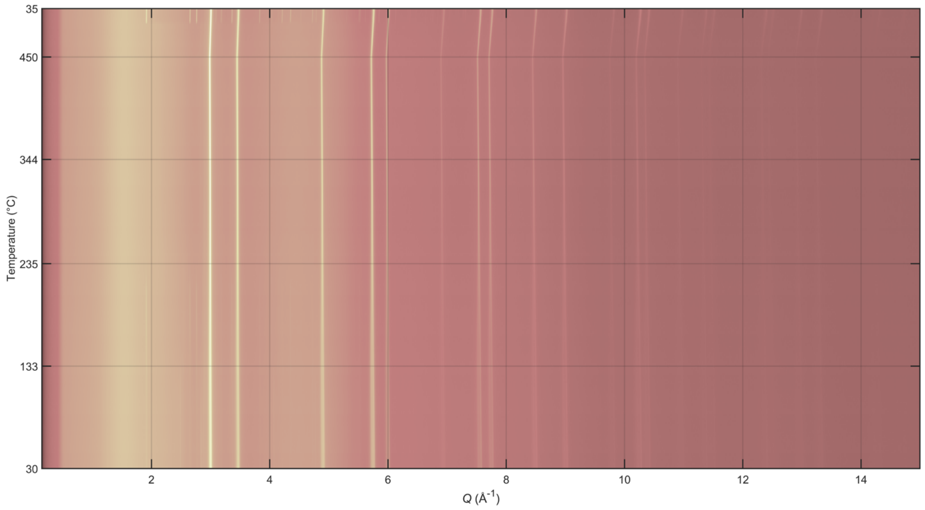

Figure S8: Contour plot of VT SPXRD data collected on the SC7 sample up to 450 °C at ALBA.

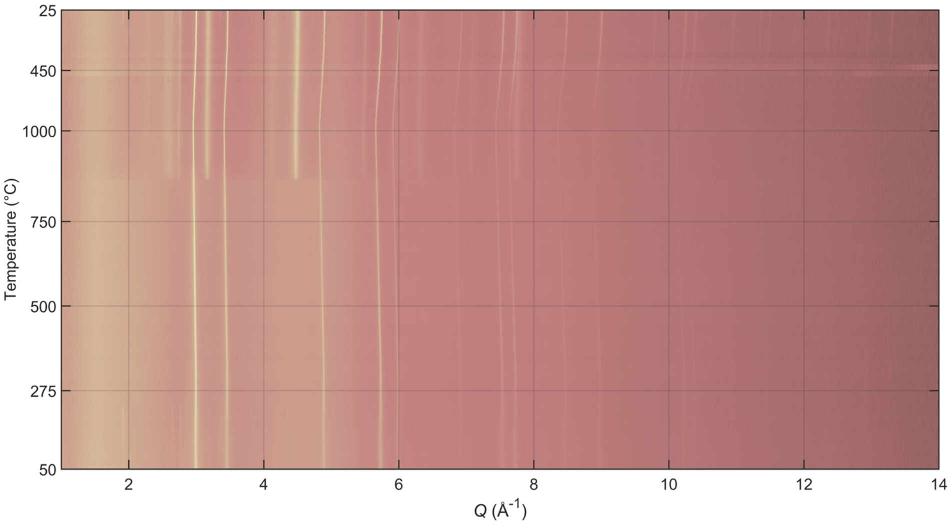

Figure S9: Contour plot of VT SPXRD data collected on the LC7 sample up to 1000 °C at ESRF. Note that at approximately 850 °C additional peaks appear due to crystallization of the alumina matrix, which remain upon cooling.

Table S4: Linear fit parameters corresponding to the fitting of the unit cell parameter of SC7 Bi-poor as function of temperature (Figure 6(b)), in the temperature range 73-238 °C.

Equation	y = a + b*x
Plot	Cell_A_ph2_pat1
Weight	Instrumental (=1/ei^2)
Intercept	3.61289 ± 2.19902E-5
Slope	7.32218E-5 ± 1.49527E-7
Residual Sum of Squares	566.83915
Pearson's r	0.99982
R-Square (COD)	0.99964
Adj. R-Square	0.99963

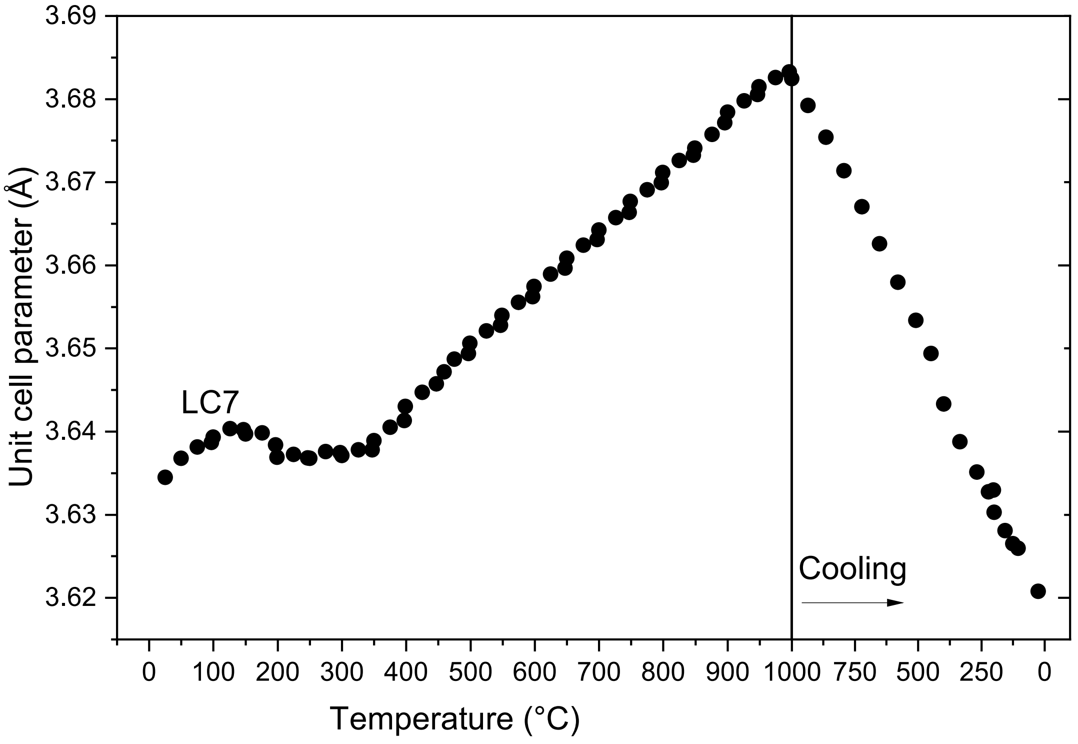

Figure S10: Refined unit cell a-parameter of the $Cu_{1-x}Bi_x$ phase in LC7 as function of temperature.

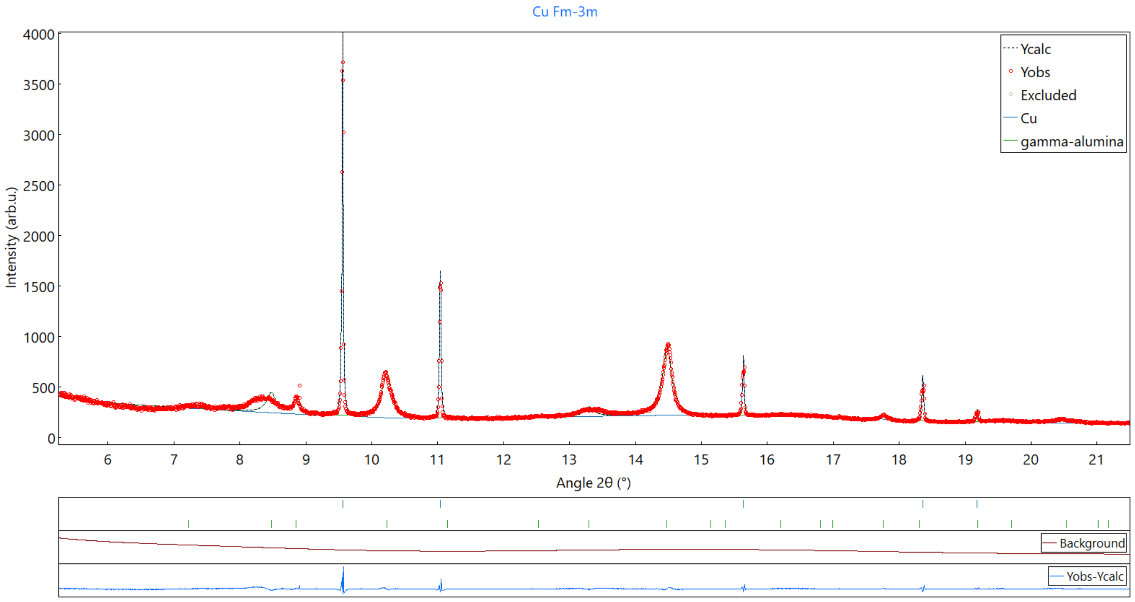

Figure S11: Rietveld refinement of SPXRD data collected on sample LC7 at 996 °C. The sharp peaks correspond to the Cu phase, whereas the broad peaks correspond to the gamma-alumina phase, refined in space group *Fm*-3*m*.

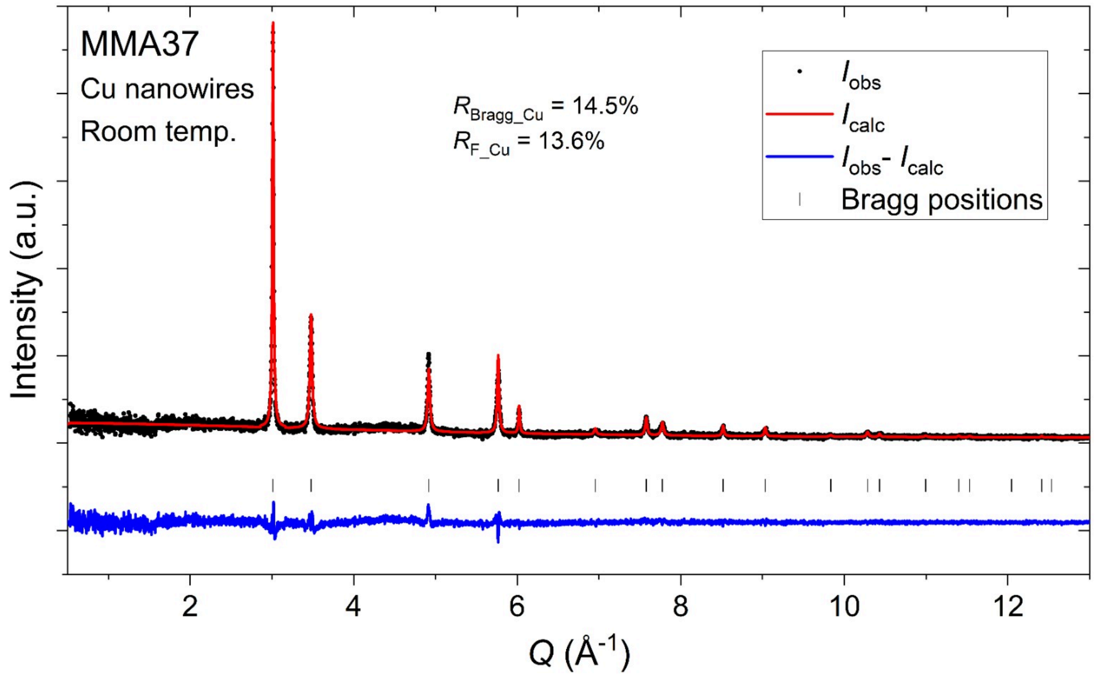

Figure S12: Rietveld refinement of room temperature TS data of pure Cu NWs ($Cu_{1-x}Bi_x$, x=0), collected at ESRF.

Released $Cu_{1-x}Bi_x$ nanowires

Figure S12 shows a contour plot of time-resolved synchrotron TS data collected on the released SC NWs sample (SC7-B_Released) before, during and after heating to 400 °C for 20 min, as well as Rietveld analysis of summed TS datasets from the three stages. The SC7-B_Released sample consists of free nanowires with nominal composition $Cu_{0.93}Bi_{0.07}$ that have been released from the alumina matrix. The observed phase evolution is very similar to that seen for the embedded sample. Again, the cubic *fcc* $Cu_{1-x}Bi_x$ is the main crystalline phase observed at all three stages with a small amount of metallic Bi present as a secondary phase, which melts when heated followed by recrystallization by a larger amount when quenched. In addition, weak peaks arising from an unknown impurity phase(s) are observed (white and black arrows). Previously reported structures of suspected impurity candidates based on the compounds used during synthesis of the NWs or removal of the alumina matrix, including Bi_2O_3, Al_2O_3, CrO_3, Cu_2O, were tested, but did not yield a match. The peaks remain constant throughout the entire experiment (heating and quenching) and can thus be concluded to be inactive and their effect negligible for the reactions related to the nanowires. Notably, none of the inactive impurities seen for the SC7-B_Released sample are observed for the embedded sample, indicating that they are most likely formed as a result of the process used for the liberation from the alumina matrix.

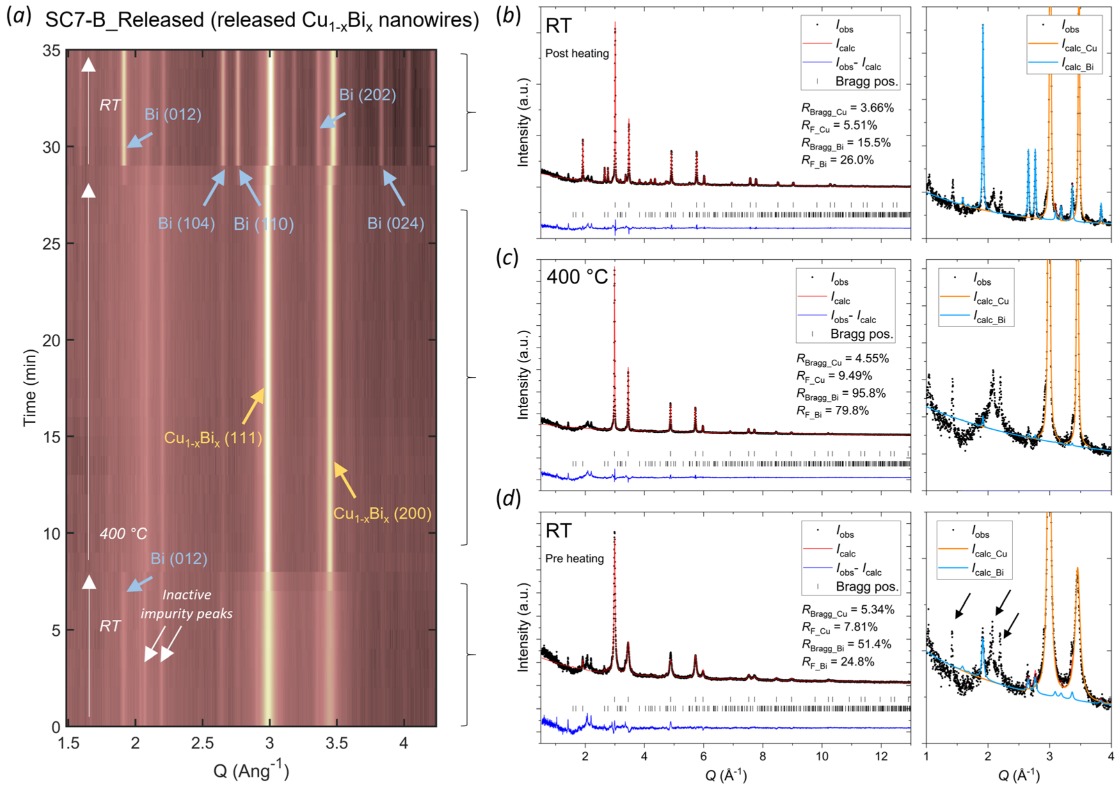

Figure S13: (a) Contour plot of low Q-range of time-resolved synchrotron TS data collected on the released SC NWs $Cu_{1-x}Bi_x$ sample (MMA55) before, during and after heating to 400 °C. (b-d) Rietveld fits of summed TS data from the three stages. The enhanced Q-regions on the right illustrate the disappearance and recrystallization of the secondary Bi phase during the experiment.

Table S5: List of low-r atomic correlations for the Cu *fcc* structure giving rise to peaks in the PDF. Potential interatomic distances for local Bi-Bi ordering suggested by the PDF data from the $Cu_{1-x}Bi_x$ NWs prior to heating are indicated by (Bi-Bi?) and coloured according to the corresponding distances illustrated in Figure 9.

**Pair**	**Distance (Å)**	**Multiplicity**
Cu-Cu	2.57	48
Cu-Cu	3.64	24
Cu-Cu **(Bi-Bi?)**	4.46	96
Cu-Cu	5.15	48
Cu-Cu	5.75	96
Cu-Cu	6.30	32
Cu-Cu **(Bi-Bi?)**	6.81	192
Cu-Cu	7.28	24
….		

(a) (b)

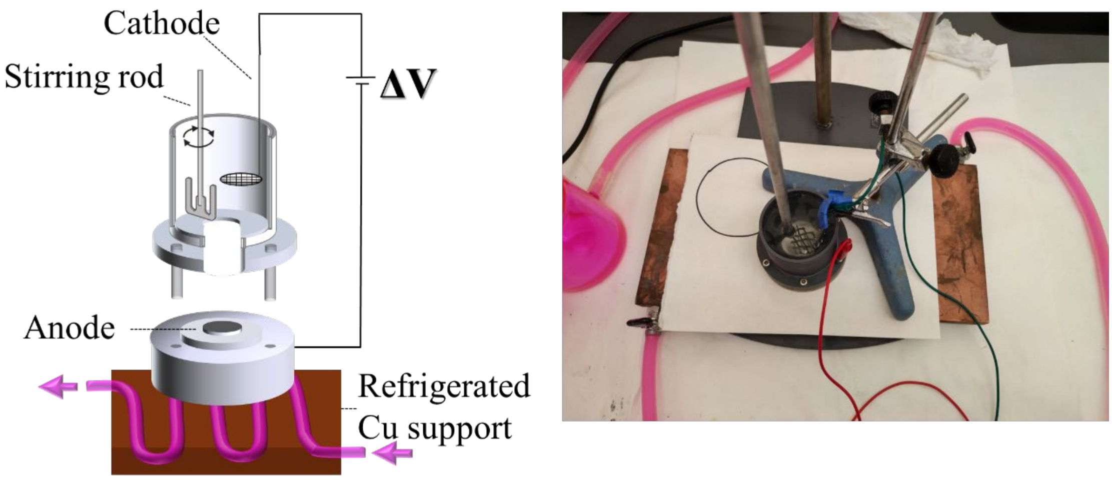

Figure S14: (a) Illustration and (b) picture of the setup used for the self-ordered porous AAO anodization at constant temperature. The setup was equipped with a Cu plate below the anodization cell connected to a circulating cryothermostat for refrigeration.
